# Analysis of interleukin-1 receptor associated kinase-3 (IRAK3) function in modulating expression of inflammatory markers in cell culture models: A systematic review and meta-analysis

**DOI:** 10.1371/journal.pone.0244570

**Published:** 2020-12-31

**Authors:** Trang Hong Nguyen, Ilona Turek, Terri Meehan-Andrews, Anita Zacharias, Helen Irving

**Affiliations:** Department of Pharmacy and Biomedical Sciences, La Trobe Institute for Molecular Science, La Trobe University, Bendigo, Victoria, Australia; Indiana University School of Medicine, UNITED STATES

## Abstract

**Background:**

IRAK3 is a critical modulator of inflammation in innate immunity. IRAK3 is associated with many inflammatory diseases, including sepsis, and is required in endotoxin tolerance to maintain homeostasis of inflammation. The impact of IRAK3 on inflammatory markers such as nuclear factor kappa-light-chain-enhancer of activated B cells (NF-κB), tumour necrosis factor-α (TNF-α) and interleukin-6 (IL-6) in cell culture models remains controversial.

**Objective:**

To analyse temporal effects of IRAK3 on inflammatory markers after one- or two-challenge interventions in cell culture models.

**Methods:**

A systematic search was performed to identify *in vitro* cell studies reporting outcome measures of expression of IRAK3 and inflammatory markers. Meta-analyses were performed where sufficient data were available. Comparisons of outcome measures were performed between different cell lines and human and mouse primary cells.

**Results:**

The literature search identified 7766 studies for screening. After screening titles, abstracts and full-texts, a total of 89 studies were included in the systematic review.

**Conclusions:**

The review identifies significant effects of IRAK3 on decreasing NF-κB DNA binding activity in cell lines, TNF-α protein level at intermediate time intervals (4h–15h) in cell lines or at long term intervals (16h–48h) in mouse primary cells following one-challenge. The patterns of TNF-α protein expression in human cell lines and human primary cells in response to one-challenge are more similar than in mouse primary cells. Meta-analyses confirm a negative correlation between IRAK3 and inflammatory cytokine (IL-6 and TNF-α) expression after two-challenges.

## Introduction

Inflammation is primarily initiated by the innate immune system as the first line of defence in response to infection or tissue injury. Infectious microbes express molecules with specific structural patterns that are known as pathogen-associated molecular patterns (PAMPs) and are recognized by pattern recognition receptors of the innate immune system, including Toll-like receptors (TLRs) [1]. While TLRs recognize exogenous PAMPs, interleukin-1 receptors (IL-1Rs) respond to endogenous cytokines such as IL-1 and IL-18, and then innate immune responses are generated through these TLR and IL-1R –induced signalling pathways [2, 3]. Both IL-1Rs and TLRs are membrane-spanning proteins and have an intracellular domain, called Toll/IL-1 Receptor (TIR) domain, which is utilized for signalling of the innate immune system [2]. Following engagement of PAMPs or cytokines with TLRs/IL-1Rs, changes in the intracellular domain of TLRs or IL-1Rs occur that enable TIR domains to bind the cytosolic TIR domain-containing adaptor protein called myeloid differentiation primary response 88 (Myd88). Myd88 forms a complex with interleukin-1 receptor associated kinase (IRAK) family members including IRAK1, IRAK2 and IRAK4, referred to as the myddosome complex [4]. Within the myddosome complex, IRAK4 activates IRAK1 or IRAK2 which are released from the complex and interact with tumor necrosis factor receptor associated factor 6 (TRAF6). TRAF6 then triggers activation of nuclear translocation of the transcription factor NF-κB [4]. In cell nuclei, NF-κB interacts with the consensus motif on promoters of many genes including inflammatory cytokine genes to induce transcription of these genes [5].

IRAK3, another member of the IRAK family, inhibits the dissociation of IRAK1 or IRAK2 from myddosome complexes and therefore the interaction of IRAK1 or IRAK2 with TRAF6, required for NF-κB activation [6–9]. Thus, IRAK3 is a key modulator of inflammatory responses. IRAK3 is mainly found in monocytes and macrophages, and is therefore also known as IRAK-M [4, 10]. IRAK3 was initially characterized as a positive regulator of the inflammatory signal cascade in which overexpression of IRAK3 enhanced NF-κB activation [10]. However, more recent studies show that NF-κB activity or inflammatory cytokine levels are increased in IRAK3 downregulation, demonstrating IRAK3 has an inhibitory role in inflammation [6, 11–13]. As one of the crucial proteins regulating pathways of innate immune signalling [6], IRAK3 has been found to be associated with many significant diseases such as sepsis [14], cancer [15], and asthma [12] and is considered a promising biomarker candidate for diagnosis of these diseases and a potential therapeutic target. Therefore, the purpose of this review is to provide a greater understanding about IRAK3 function in inflammation based on systematic analysis of quantitative data reported in published studies until now.

Importantly, IRAK3 is involved in regulation of endotoxin tolerance [6, 16, 17]. Endotoxins are toxins found in bacteria and include compounds such as lipopolysaccharide (LPS), an outer membrane component of Gram-negative bacteria and a prototypical PAMP. Endotoxin tolerance is a mechanism used to maintain homeostasis and prevent uncontrolled inflammation. In endotoxin tolerance, cells or organisms exposed to endotoxins, enter into an unresponsive state and are unable to respond to further endotoxin stimulation, preventing an inflammatory overload [18, 19]. Moreover, endotoxin tolerance is related to sepsis pathophysiology. Sepsis is an inflammatory disease and consists of two phases. The first phase is characterized by hyper-inflammatory responses to severe infections. The second phase of sepsis happens later or simultaneously, and is characterized by immunosuppression caused by endotoxin tolerance [18, 19]. Endotoxin tolerance can be accompanied by patient susceptibility to secondary infections. IRAK3 is required for endotoxin tolerance in macrophages, as IRAK3 deficiency causes elevated pro-inflammatory cytokine production compared to wild-type macrophages upon LPS re-challenge, thereby showing a lack of endotoxin tolerance [6, 11, 17]. IRAK3 is also involved in regulating endotoxin tolerance in dendritic cells [20] and in *in vivo* rat model [16].

IRAK3 mRNA and protein expression and its effects on inflammation have been studied widely using various experimental models including cell lines [10, 13, 21–24], human or mouse primary cells [25–28], and animal *in vivo* models [29–31], and reported to have contradicting effects on immune responses. Wesche *et al*. [10] reported overexpression of IRAK3 in cells results in increased level of NF-κB activity after challenge with LPS. IRAK3 may induce NF-κB activity in response to IL-1β stimulation [8, 10]. In dendritic cells challenged with IL-33, IRAK3 also functions to activate the expression of inflammatory cytokines including IL-6, IL-5 and IL-13 [9]. Kim *et al*. [32] reported IRAK3 overexpression in macrophages requires IRAK1 knock-down to suppress NF-κB activation and cytokine TNF-α production. Cole *et al*. [33] found IRAK3 has no inhibitory role on TNF-α protein production in mouse bone marrow derived dendritic cells. However, in mouse macrophages IRAK3 knockout resulted in increased levels of inflammatory cytokines (IL-6 and TNF-α) [6]. Additionally, Zhou *et al*. [7] suggested an alternative regulatory pathway in which the interaction of IRAK3 with myddosome complexes induces cascade of mitogen activated protein kinase kinase kinase 3 (MEKK3) dependent NF-κB activation. NF-κB activation then triggers transcription of anti-inflammatory molecules, which generates an overall inhibitory effect on inflammatory responses [7]. Furthermore, IRAK3 contains a catalytic guanylate cyclase centre which can generate a nano-environment of cyclic guanosine monophosphate surrounding IRAK3 protein that appears to be involved in the modulatory mechanism of IRAK3 effect on NF-κB activation [34]. The various actions of IRAK3 on inflammation appear to be affected by ligand specificity and dose [35], or cell types [33].

**Fig 1:**
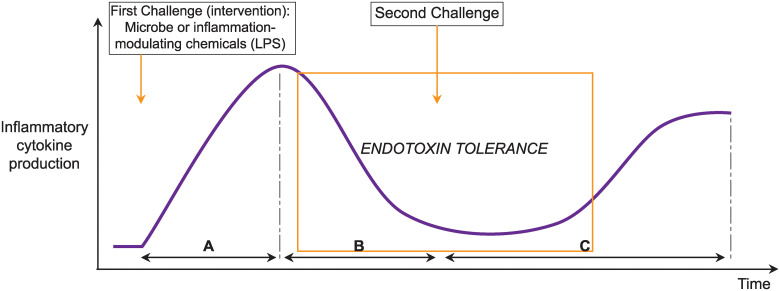
Scheme showing predicted changes in inflammatory cytokine production upon one- and two-challenges by microbes (bacteria or protozoa) or inflammation-modulating chemicals. After a temporal duration (A) following first challenge by a microbe or inflammation-modulating chemical, inflammatory cytokine production is markedly increased, and then downregulated by intracellular inhibitors including IRAK3 to maintain homeostasis of inflammation responses for an interval of time (B). The phenomenon in which levels of inflammatory cytokines are limited by these intracellular inhibitors (e.g. IRAK3) is called endotoxin tolerance (depicted by the rectangle). Upon a second challenge the homeostatic effect of negative inflammatory regulators, including IRAK3, remains significant and reduces production of inflammatory cytokines for another temporal period (C). The temporal periods are uncertain and are analysed in this review.

Considering the multiple pathways implicated in IRAK3 action, the research questions of this review are to identify to what extent IRAK3 has inhibitory effects on inflammation, and how the actions of IRAK3 are temporally regulated. The function of IRAK3 in inflammation has been the subject of a number of research studies [6, 7, 10, 13] and narrative reviews [4, 36]. However, to our knowledge, a systematic review or meta-analysis has not been conducted to date. This systematic review and meta-analyses aim to elucidate if changes in levels of inflammatory markers can be correlated to temporal changes in IRAK3 expression and its effects in regulating inflammatory responses in cell culture models. In addition to NF-κB activity, the inflammatory markers investigated in this review are TNF-α and IL-6. Both TNF-α and IL-6 are pro-inflammatory cytokines, and IL-6 can also function as an anti-inflammatory cytokine [37]. The anti-inflammatory effects of IL-6 are activated under certain situations, and mostly occur in IL-6 classic signalling which is initiated by the binding of IL-6 to membrane bound or soluble IL-6Rs [38, 39]. In pro-inflammatory response to pathogenic infections, TNF-α induces vasodilation and permeability to allow the mobility of immune cells to the sites of damage or infection, and IL-6 induces complement and opsonisation [40]. Whereas elevation of TNF-α occurs for a short period of time in the initial phase of sepsis, the increased levels of IL-6 occur for longer periods and are associated with increased mortality in septic patients [41]. Specifically, we aimed to analyse protein and mRNA expression of IRAK3 and its impact on the changes of inflammatory marker levels following specific temporal periods after challenge intervention (Fig 1). The two types of interventions involved in this review are one-challenge and two-challenge interventions. In the one-challenge intervention, cells are subjected to the treatment with a microbe or an inflammation-modulating chemical, and the outcomes measured at specific periods of time after the challenge. In the two-challenge intervention, cells are subjected to the treatment with a microbe or an inflammation-modulating chemical; and at a specific period of time after the first challenge cells are treated again with the same or different microbe or chemical, and the outcomes measured at specific periods of time after the second challenge.

## Method

### Search strategy and identification of studies

Six databases (Scopus, Web of Knowledge, Medline, ScienceDirect, Embase, PubMed) were searched systematically from the earliest date available until 16 July 2020. The search keywords were conducted using three concepts: IRAK, endotoxin tolerance, and sepsis. Synonyms within each concept were combined using the OR Boolean operator (IRAK or interleukin-1 receptor associated kinase, endotoxin tolerance or lipopolysaccharide tolerance).

Titles and abstracts were screened initially by one reviewer (T.N.) using selection criteria described in Table 1. Selected studies from the first screening were then screened independently by two reviewers (T.N. and H.I./I.T./T.M.-A.). The full text of the studies selected based on the title and abstract screening, was verified independently by two reviewers (T.N. and H.I./I.T./T.M.-A.) using the same selection criteria. The reviewers discussed differences in opinion until consensus was reached.

**Table 1:** Study selection criteria.

Criteria
Must contain the concept of inflammation, regulation or intervention of inflammation.
Must contain the concept of IRAK / lipopolysaccharide tolerance / endotoxin tolerance / sepsis.
Must be mammalian species studies.
Must be peer-reviewed research studies.
Must use cell culture models.
Must contain at least one of the outcomes (IRAK3 mRNA and protein expression, NF-κB activity, TNF-α and IL-6 protein levels).
Must be in English.

### Population

The included studies were restricted to animal or human cell culture studies. Cell culture models used in the studies are cell lines, human primary cells and mouse primary cells.

### Intervention

The animal or human cell culture models were challenged by bacteria (*Mycobacterium tuberculosis*, *Escherichia coli*, *Streptococcus pneumoniae*, *Haemophilus influenzae*, *Lactobacillus acidophilus*), protozoa (*Leishmania donovani* promastigotes) or inflammation-modulating chemicals (e.g. LPS, *Helicobacter pylori* antigen, heat-stable enterotoxigenic *E*. *coli* pathogen-associated molecular patterns, Pam3CysSerLys4 (Pam3CSK4), lipoteichoic acid, peptidoglycan, IL-1, S-nitrosoglutathione, transforming growth factor beta (TGF-β), paramethoxyamphetamine, vitamin D3, 6-methylprednisolone, surfactant protein A, titanium particles/bone cement, granulocyte-macrophage colony-stimulating factor (GM-CSF)/ interferon gamma (IFN-ϒ), IL-13, hyperoxia (95% O_2_), TLR7 agonist (R837, R848, 1V136), IL-1β, TNF-α). Descriptions of the intervention chemicals can be found in S1 Table (S1 File).

### Comparisons

There are three types of comparisons: comparisons between a control group which was treated with phosphate-buffered saline or sterile water and a group receiving one-challenge as described above; comparisons between a group receiving one-challenge and a group receiving two-challenges as described above; and comparisons between an IRAK3 silencing/knockout group and an IRAK3 present group.

### Outcomes

Outcomes that are measures of IRAK3 mRNA and protein expression, inflammatory cytokine (TNF-α or IL-6) protein production and NF-κB activity were included.

### Research design

The articles must be research studies containing cell culture models. Review articles and *in vivo* studies were excluded.

### Data extraction

Data including cell type, study design, description of intervention, intervention duration and outcome measure were extracted from each included study (S2 Table in S1 File). Data extraction was performed by one author (T.N.) and verified by a second author (either H.I., T.M.-A. or I.T.). In cases where there were no numerical data provided in the studies, the data were estimated from graphs. If there were insufficient data in the studies, data were requested from the original author or qualitatively assessed if not provided. The relevant data were extracted and entered directly into Review Manager (RevMan) Version 5.3 by one author (T.N.) and checked by a second author (H.I./T.M.-A./I.T./A.Z.).

### Data synthesis

The studies are grouped for meta-analyses based on population, intervention and outcome measures of interest, which can be found in Table 2. Separate meta-analyses were conducted for studies involving cell lines, mouse primary cells and human primary cells, as adaptation of cell lines to culture environments may differ genetically and phenotypically from their tissue origin, while primary cells reflect more specifically *in vivo* phenotypes. Moreover, primary cells are classified into human and mouse groups due to species differences in immune responses [42]. Interventions are also grouped as challenges by microbes (bacteria or protozoa) and bacteria-derived compounds (LPS, heat-stable enterotoxigenic *E*. *coli* pathogen-associated molecular patterns Pam3CSK4, lipoteichoic acid, *Helicobacter pylori* antigen and peptidoglycan) in one group, and other challenge chemicals are in separate groups. RevMan 5.3 software was used to perform the meta-analyses. Standardized mean difference (SMD) and 95% confidence intervals (CIs) were calculated for outcome measures of interest to indicate the effect size. The I^2^ statistic was used for assessing heterogeneity [43]. A value of 0% was interpreted as indicating no observed heterogeneity, and a value of 100% was considered to be a completely heterogeneous sample. Values of 25%, 50% and 75% indicated low, moderate and high levels of heterogeneity [43].

**Table 2:** Basis for comparisons by meta-analysis.

Cell culture models	Durations of outcome measurement after intervention	Comparison groups	Outcome measures
Cell lines	Short term (ST) (5min– 3h)	Control versus One-challenge	IRAK3 mRNA expression
Mouse primary cells	Intermediate term (IT) (4h–15h)	One-challenge versus Two-challenges	IRAK3 protein expression
			TNF-α protein level
Human primary cells	Long term (LT) (16h–48h)	IRAK3 silencing/ knockout versus IRAK3 present	IL-6 protein level
	Short term NF-κB (ST_N_) (5min– 30min)		NF-κB activity
	Intermediate term NF-κB (IT_N_) (31min– 5h)		
	Long term NF-κB (LT_N_) (6h–24h)		

As durations of outcomes measured after interventions are differently classified for NF-κB activity, the abbreviations of short term, intermediate term and long term of NF-κB activity have added _N_ subscript (ST_N_, IT_N_ and LT_N_).

## Results

### Yield

**Fig 2:**
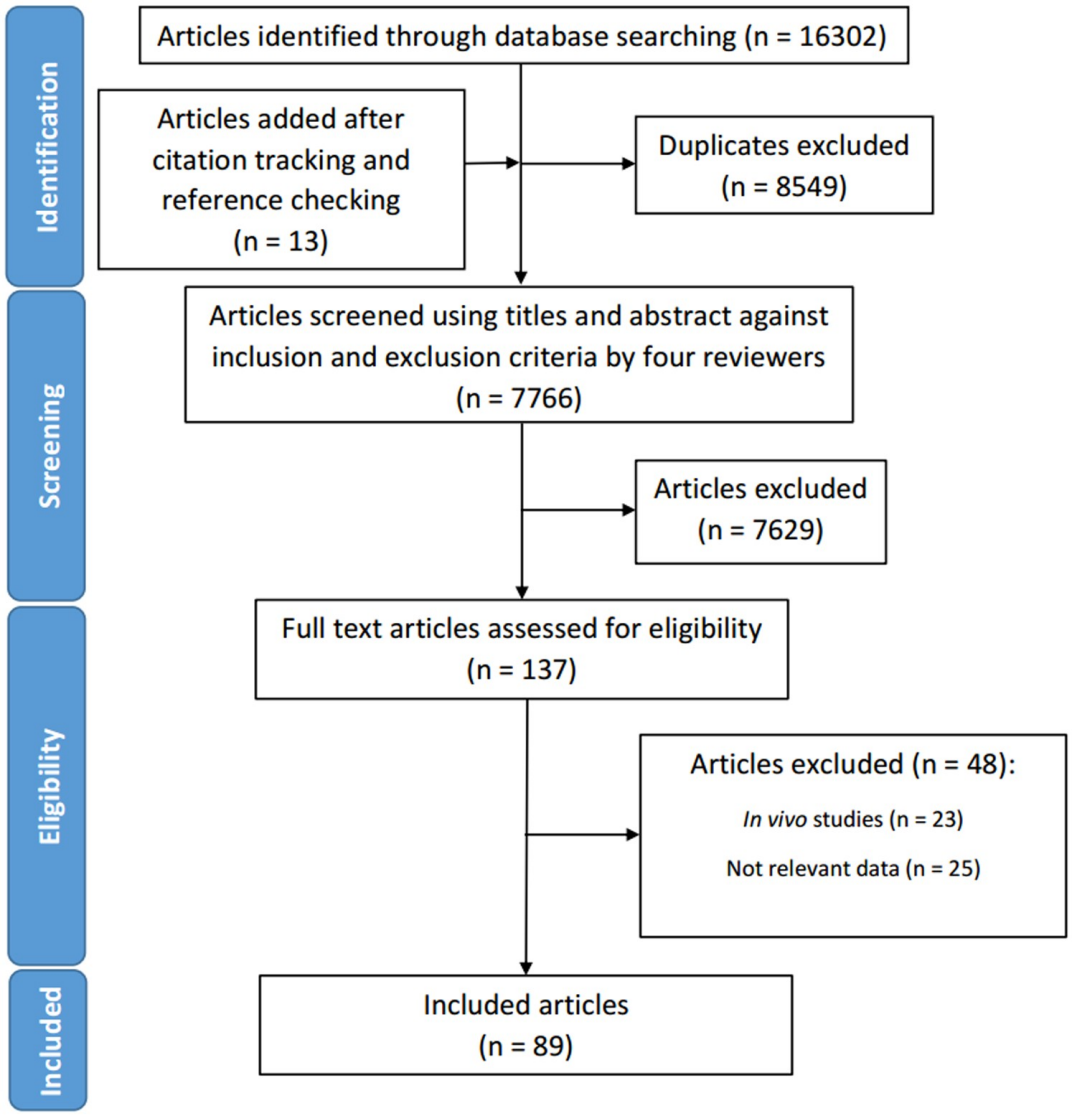
PRISMA flowchart depicting the process of search and selection of studies for the systematic review.

A total of 16,302 studies were identified through database searches, and 8,549 duplicate studies were removed (Fig 2). Citation tracking and reference checking added an additional 13 studies (Fig 2). The studies underwent screening of titles and abstracts based on study selection criteria (Table 1), and as a result 7,629 studies were excluded and 137 studies were selected. After full-text screening, 89 studies were included in the systematic review. Studies excluded from full-text screening and the reasons for exclusion can be found in S3 Table (S1 File).

### Characteristics of included studies

Of the 89 included studies, 31 used cell lines^1^, 28 used human primary cells^2^, 25 used mouse primary cells^3^, two used cell lines and mouse primary cells [13, 44], one used human and mouse primary cells [45], one used cell line and human primary cells [46], and one used cell line, mouse primary cells, human cells [47]. Most studies (65 studies) utilized challenges with LPS (1 ng– 100 μg/mL) (three studies used *E*. *coli* and *Porphyromonas gingivalis* LPS, one study used *E*. *coli* and *Salmonella minnesota* LPS, 33 studies used LPS from *E*. *coli*, two used LPS from *P*. *gingivalis*, five studies used *Salmonella abortus* LPS, one used *Klebsiella* LPS, one used *Aggregatibacter actinomucetencomit* LPS, two used LPS from *S*. *minnesota*, one used *Salmonella enteritidis* LPS, 16 studies did not mention the bacterial origin of LPS); three studies used Pam3CSK4; three studies used TLR7 ligand; three studies used IL-1β; eight studies used bacteria; one study used protozoa; two studies used TNF-α; several single studies used challenges with one of these chemicals (heat-stable enterotoxigenic *E*. *coli* pathogen-associated molecular patterns, lipoteichoic acid, peptidoglycan, *Helicobacter pylori* antigen, IL-1, S-nitrosoglutathione, TGF-β, paramethoxyamphetamine, vitamin D3, 6-methylprednisolone, surfactant protein A, titanium particles/bone cement, GM-CSF/IFN-ϒ, IL-13, hyperoxia (95% O_2_).

^1^ References [8, 10, 12, 21–24, 32, 48–70].

^2^ References [15, 17, 25–28, 71–92].

^3^ References [6, 7, 11, 20, 33, 35, 93–111].

Most studies (89 studies) performed one-challenge and measured outcomes of interest from 30min to 48h post-treatment; one study involved five days (5d); one study used 6d; one study reported data from 3d and 4d post-treatment; and three studies involved less than 30min (5 to 15min). The interval of less than 3h is for measuring outcomes involving nuclear translocation and DNA binding of transcription factors such as NF-κB. In time intervals less than 3h, there is no significant increase of IRAK3 mRNA and protein expression [22, 50, 55]. From 4h to 15h IRAK3 mRNA and protein levels increased significantly [13, 22, 45, 55, 76]. The periods of outcome measurement after one-challenge used most frequently were from 16h to 48h in experiments of silencing or knockout of the IRAK3 gene; these studies also reported the impact of IRAK3 on inflammation during this interval (16h–48h) [6, 13, 22, 60, 96, 97, 105]. Therefore, for the purposes of this review, following one-challenge intervention, durations of outcome measurement were classified into short term (ST) (5min– 3h), IT (intermediate term) (4h–15h) and LT (long term) (16h–48h). Two-challenge results were reported in 32 studies. Thirty one studies performed first challenge for periods between 1h and 48h, one study involved 5d [90], and after second challenge outcomes were measured between 5min and 48h. The characteristics of the included studies can be found in S2 Table (S1 File).

### Intervention: One-challenge

This review analyses IRAK3 mRNA and protein expression, NF-κB activity, TNF-α and IL-6 protein levels in cell lines, human or mouse primary cells at ST, IT and LT after intervention of one-challenge with microbes or inflammation-modulating chemicals. As mentioned above, interventions are grouped for meta-analyses, with challenges by microbes (bacteria or protozoa) and bacteria-derived compounds (LPS, heat-stable enterotoxigenic *E*. *coli* pathogen-associated molecular patterns, Pam3CSK4, lipoteichoic acid, *H*. *pylori* antigen and peptidoglycan) included in one group, and other inflammation-modulating chemicals in separate groups. Changes in NF-κB activity, IRAK3 and cytokine expression levels are analysed by comparing control cells as described above with cells receiving one-challenge. The impact of IRAK3 at ST, IT or LT temporal periods on cytokine expressions is examined by comparing wildtype cells and cells with IRAK3 silenced or knocked out.

#### Effect of one-challenge intervention on IRAK3 mRNA expression

**Fig 3:**
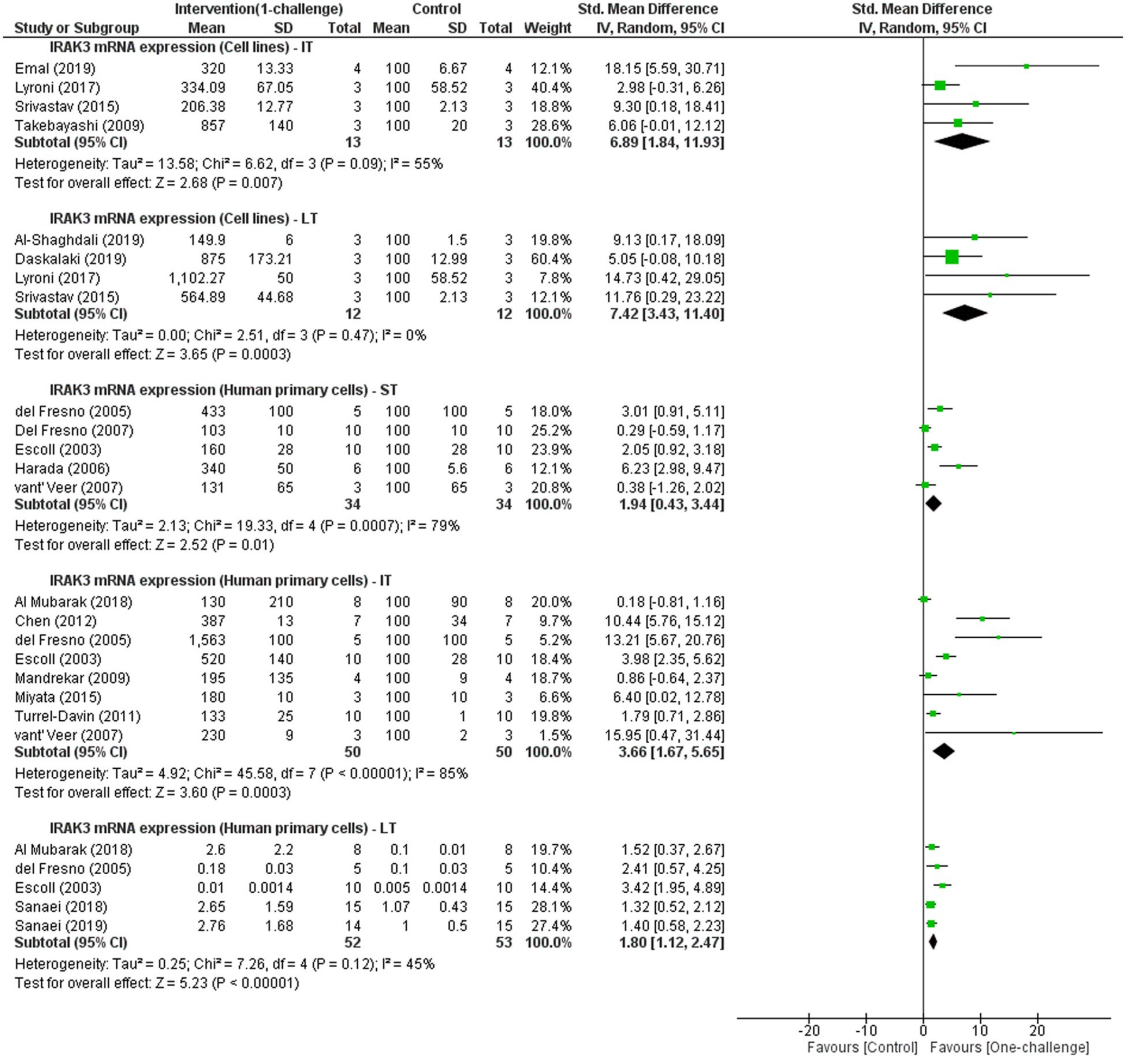
IRAK3 mRNA expression outcome in cell lines and human primary cells: One-challenge intervention group versus control group. Intervention was performed using LPS [17, 23–25, 27, 28, 46, 47, 55, 61, 76, 77, 84, 88, 92, 93], *L*. *donovani* [13] or *H*. *influenzae* [81]. IRAK3 mRNA expression was measured at short term (ST; 5min– 3h), intermediate term (IT; 4h–15h) and long term (LT; 16h–48h) after one-challenge.

Meta-analyses of IRAK3 mRNA expression in cell lines identify significant increases at IT and LT after one-challenge with LPS or *L*. *donovani* (Fig 3). Two studies using mouse cell lines (RAW 264.7) reported different findings of IRAK3 mRNA expression at ST; one study showed IRAK3 mRNA expression trended to increase following one-challenge with *L*. *donovani* compared to control group [13], whereas other reported no increase of IRAK3 mRNA expression upon LPS challenge [55]. Meta-analysis of IRAK3 mRNA expression in human primary cells challenged with LPS or *H*. *influenzae* shows a significant increase at ST, IT and LT (Fig 3). Meta-analyses of data from one-challenge with LPS, *L*. *donovani* or *S*. *pneumoniae* show there was no significant change in IRAK3 mRNA at ST and LT after one-challenge, but a significant increase at IT in mouse primary cells (S1 Fig in S2 File).

Eleven studies were not suitable for meta-analysis because they differed in their intervention [15, 57, 64, 75, 82, 85], or included incomplete data for meta-analysis [22, 45, 68, 106], or measured outcomes outside the investigated range (5d) [94]. Ho *et al*. [68] reported one-challenge with LPS markedly increased IRAK3 mRNA expression at 3h (ST) in intestinal porcine enterocyte cell line. Domon *et al*. [22] reported increases of IRAK3 mRNA expression at IT (from 9h to 12h) after LPS stimulation in macrophages derived from monocytic cell line, and these results are similar to results of the meta-analysis of cell line studies conducted in this review (Fig 3). Gunthner *et al*. [45] reported IRAK3 mRNA levels in human and mouse peripheral blood mononuclear cells was significantly induced at IT and LT (from 4h to 24h) after LPS challenge, similar to the meta-analyses (Fig 3). Stiehm *et al*. [106] using mouse bone marrow dendritic cells reported maximum up-regulated IRAK3 mRNA levels occurred at 24h (LT) and plateaued until 48h after LPS challenge, which differs from the results of the meta-analysis showing no significant increase of IRAK3 mRNA levels at LT (S1 Fig in S2 File). Geng *et al*. [94] reported reduction in IRAK3 mRNA level at 5d after treatment of mouse bone marrow monocytes with sub-nanomolar concentration of LPS. Five studies reported significant inductions in IRAK3 mRNA levels after one-challenge with S-nitrosoglutathione at 16h (IT) [75], TNF-α at 2h (ST) [75], surfactant protein A which is a lung collectin having inhibitory effects on TLR4 signalling at 12h (IT) [82], vitamin D3 from 1d to 6d [57], TGF-β from 4h to 24h (IT and LT) [15], and titanium particles/bone cement at 6h (IT) [64]. Although IRAK3 mRNA expression was induced in the treatment with a glucocorticoid (6-methylprednisolone) in human monocytic (THP-1) and mouse macrophage (RAW264.7) cell lines, IRAK3 mRNA expression was decreased at 6d after 6-methylprednisolone treatment in osteoclast-like cells differentiated from THP-1 or RAW264.7 [57]. Another study reported IRAK3 mRNA expression tends to decrease at 2h and 8h (ST and IT) after treatment with IL-13 [85]. Peak increase of IRAK3 mRNA expression was reached at 24h (LT) in mouse macrophage cell line and primary cells challenged with *L*. *donovani* or LPS [13, 55], whereas the peak increases were reported at 6h (IT) in human monocytes challenged with LPS [25, 76].

#### Effect of one-challenge intervention on IRAK3 protein expression

**Fig 4:**
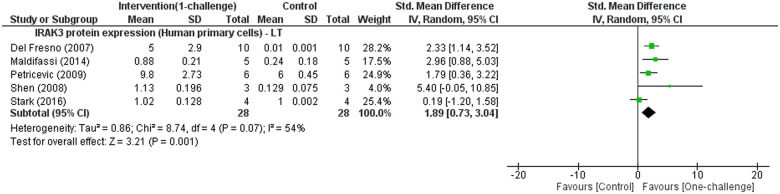
IRAK3 protein expression outcome for human primary cells: One-challenge intervention group versus control group. Intervention was performed using LPS [27, 80, 83, 86, 87]. IRAK3 protein expression was measured at long term (LT; 16h–48h) after one-challenge.

Meta-analysis identifies significant increases in IRAK3 protein expression at LT in human primary cells (Fig 4). Two studies using human cell line (Caco-2/TC7) or mouse cell line (RAW 264.7) investigated IRAK3 protein expression at ST. One study reported IRAK3 protein expression trended to increase in a mouse cell line challenged with *L*. *donovani* compared to control group [13], while another showed no increase in human cell line challenged with enterotoxigenic *E*. *coli* [50]. Additionally, two studies using mouse cell lines (RAW 264.7) reported significant increases of IRAK3 protein expression at IT and LT following one-challenge with *L*. *donovani* or LPS [13, 55].

Six studies could not be included in the meta-analysis as they differed in their interventions [62, 75], or the outcome measurements were reported only as immunoblots [58, 95], or they included incomplete data for meta-analysis [22, 91]. Domon *et al*. [22] reported no significant increase in IRAK3 protein levels at ST in a monocytic cell line, and significant induction at IT (at 9h and 15h) after challenge with *P*. *gingivalis* LPS, but no significant increase at IT after challenge with *E*. *coli* LPS. These findings of *E*. *coli* LPS challenge are similar to that identified in the current meta-analysis (Fig 4). Im *et al*. [91] reported a 1.5-fold induction of IRAK3 protein expression at 3h following one LPS challenge in human periodontal ligament cells. Su *et al*. [58] demonstrated constitutive presence of IRAK3 protein in monocytic cell lines, and no acute increase after Pam3CSK4 challenge. Two studies reported significant increases in IRAK3 protein expression after treatments with S-nitrosoglutathione at 20h (LT) in human monocytes [75], and paramethoxyamphetamine (an activator of protein kinase C playing a role in activating TLR4/IRAK1 pathway) at 6h and 12h (IT) in a monocytic cell line [62]. Using immunodetection, Hayashi *et al*. [95] showed an increase in IRAK3 protein expression at 2h (ST) and 24h (LT) after challenge with TLR7 ligand in mouse primary cells. Srivastav *et al*. [13] reported IRAK3 protein expression reached to peak increase at 24h (LT) in both mouse macrophage cell line and primary cells challenged with *L*. *donovani*, which was similar to mRNA expression.

#### Effect of one-challenge intervention on inflammatory markers

The NF-κB module consists of five NF-κB subunits which can dimerize to form up to 15 unique dimeric transcription factors [5]. Once TLRs or IL-1Rs recognize their ligands and trigger signalling cascades, the dimeric NF-κB transcription factor is activated to translocate from the cytoplasm into the cell nucleus and interacts with the consensus motif found in promoters of inflammatory genes. There are several ways to evaluate NF-κB activities such as movement of the transcription factor, DNA binding activity of NF-κB, and NF-κB reporter gene expression assay. Time scales of these methods differ enormously, as the nuclear translocation of NF-κB transcription factors can be detected within 30min after challenge, while NF-κB reporter gene expression is usually detected at 6h or longer after challenge. Hence this study classifies duration of outcomes measured after challenge for NF-κB activation differently from other outcomes of interest, which includes three terms: short term—ST_N_ from 5min to 30min, intermediate term—IT_N_ longer than 30min and up to 5h, and long term—LT_N_ from 6h to 24h. NF-κB activities were classified into DNA binding activity of NF-κB and NF-κB reporter gene expression.

Two cell line studies reported significant increase of DNA binding activity of NF-κB at LT_N_ after one-challenge with LPS or Pam3CSK4 [12, 22]. Meta-analysis of NF-κB reporter gene expression in cell lines also demonstrates its significant induction at LT_N_ after one-challenge (S2 Fig in S2 File). Six studies are not included in the meta-analysis, as they differed in their interventions [52, 56, 62] or measured NF-κB DNA binding activity or NF-κB reporter gene expression in human or mouse primary cells [77, 78, 109]. Odoms *et al*. [56] reported no significant increase in DNA binding activity of NF-κB from 30min (ST_N_) to 3h (IT_N_) after challenge with hyperoxia (95% O_2_) but significant elevation after IL-1β challenge in a lung epithelial cell line. Tiwari *et al*. [62] reported 7-fold induction in NF-κB reporter gene expression observed from 6h to 12h (LT_N_) in monocytic cell lines when challenged with paramethoxyamphetamine. Harada *et al*. [77] reported NF-κB DNA binding activity increased significantly in human intrahepatic biliary epithelial cells at 24h (LT_N_) post-treatment with LPS or Pam3CSK4. NF-κB reporter gene expression was found to increase markedly at 6h (LT_N_) after LPS challenge in bone marrow-derived macrophages [109], or at 24h (LT_N_) after lipoteichoic acid challenge in human periodontal ligament cells [78], or at 18h (LT_N_) after challenge with TLR7 ligand in single study involving a macrophage cell line [52].

**Fig 5:**
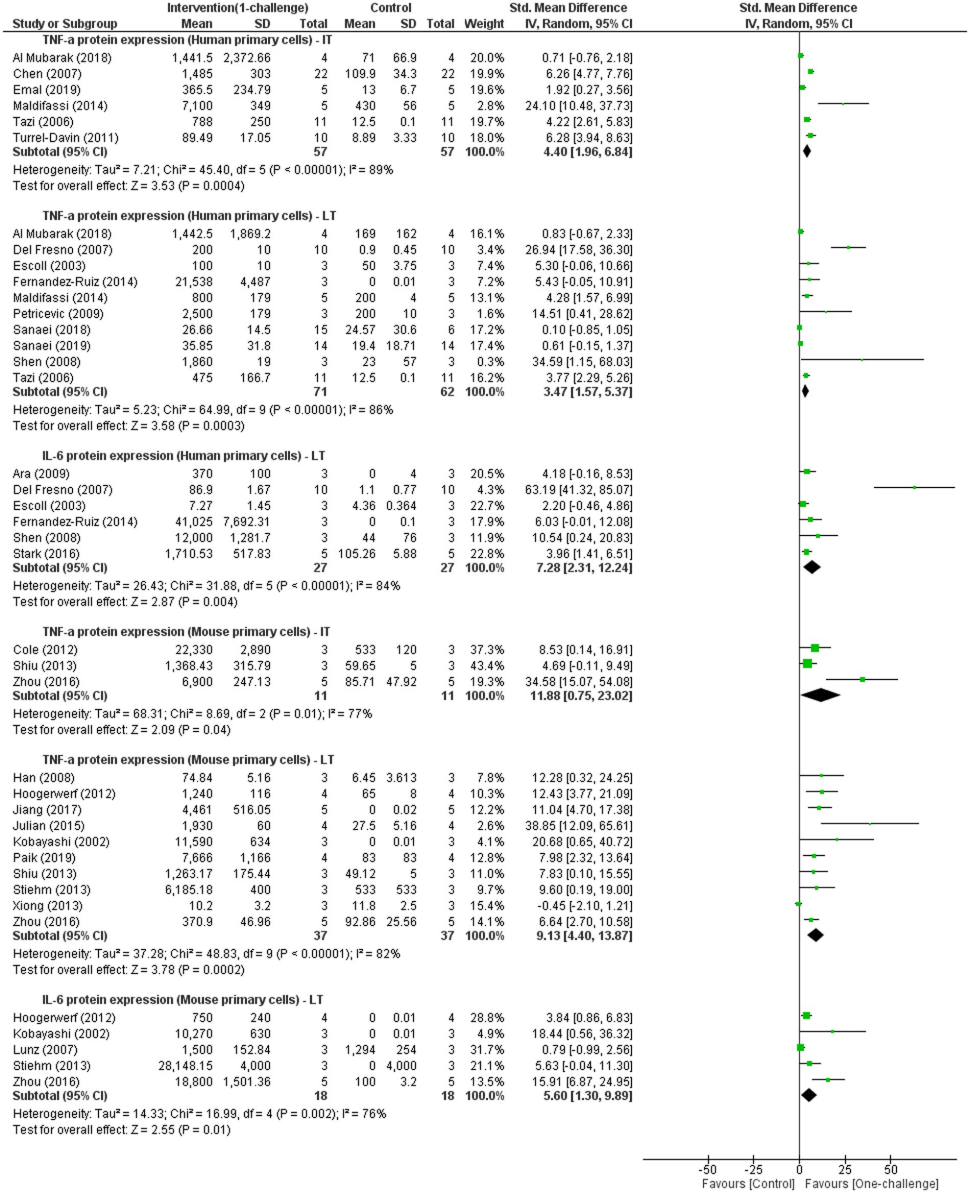
TNF-α and IL-6 protein expression outcomes for human and mouse primary cells: One-challenge intervention group versus control group. Intervention was performed using LPS [6, 25–28, 33, 35, 44, 47, 73, 74, 80, 83, 84, 86–88, 90, 92, 96–98, 102, 106, 109, 111] or *H*. *pylori* antigen [105]. TNF-α and IL-6 protein levels were measured at intermediate term (IT; 4h–15h) and long term (LT; 16h–48h) after one-challenge.

IRAK3 is a negative regulator of signalling pathways inducing production of inflammatory cytokines including TNF-α and IL-6 [6, 7]. Expression of TNF-α and IL-6 proteins is commonly reported as a measure of inflammation in cell culture studies. Meta-analyses of TNF-α protein level in cell lines, human and mouse primary cells challenged with LPS, Pam3CSK4, peptidoglycan, *L*. *donovani* or *H*. *pylori* antigen indicate that TNF-α protein expression is highly increased at IT and LT (Fig 5 and S3 Fig in S2 File). Two studies using the human cell line (THP-1) reported TNF-α protein expression significantly increased at ST after LPS challenge [48, 53]. Four studies are not included in the meta-analysis of TNF-α protein level, as they differed in their interventions [7] or included incomplete data for meta-analysis [72, 89, 110]. Almeida *et al*. [72] showed a 5-fold induction in TNF-α protein expression in human monocytes and 2.5-fold induction in human peripheral blood mononuclear cells at 24h (LT) after challenge with *M*. *tuberculosis*. Wiersinga *et al*. [89] reported a significant induction of TNF-α protein levels measured at 4h (IT) in human whole blood when challenged with LPS. LPS challenging in mouse macrophages induced LPS concentration-dependent increases in TNF-α protein levels measured at 24h (LT) [110]. Zhou *et al*. [7] reported a significant increase in TNF-α protein level from 1h (ST) to 24h (LT) in mouse bone marrow-derived macrophages after TLR7 ligand challenge. Thus, all single studies reported TNF-α protein increases in similar periods to the results of meta-analyses performed on TNF-α level in cell line, human or mouse primary cell-based studies.

IL-6 production is induced upon microbial stimulation on TLR/IL-1R signal cascade, and IL-6 plays role as a pro-inflammatory cytokine. IL-6 also has regenerative or anti-inflammatory actions which is most likely dependent on membrane bound or soluble IL-6Rs signal pathway induced by the engagement of IL-6 [38]. In this review, all included studies reported IL-6 as pro-inflammatory cytokine generated via TLRs/IL-1R pathways under microbial challenges. Two studies using human (THP-1) or mouse (BV2) cell lines reported IL-6 protein levels significantly increased at ST after LPS challenge [48, 112]. Meta-analysis of IL-6 protein level identifies significant increases at IT and LT upon LPS challenge in cell lines, and at LT in human and mouse primary cells (Fig 5 and S3 Fig in S2 File). Similarly, two studies using mouse primary cells also showed significant increase of IL-6 protein level at IT following LPS stimulation [35, 107]. Four studies are not included in the meta-analysis because they differed in their interventions [7, 79, 99], or were the only human primary cell study reporting IL-6 protein levels at IT [89]. Lee *et al*. [79] reported significant increases in IL-6 protein levels from 48h to 96h (LT) after challenge with TNF-α in human fibroblast-like synoviocytes. Wiersinga *et al*. [89] showed a significant increase in IL-6 protein levels at 4h (IT) in human whole blood when stimulated with LPS. Kanakaraj *et al*. [99] demonstrated an increasing trend in IL-6 protein production in mouse skin and embryonic fibroblasts at 8h (IT) after challenge with IL-1. Zhou *et al*. [7] showed significant induction of IL-6 at 1h, 4h, 12h and 24h (ST, IT, LT) upon TLR7 ligand challenge, with approximately 10-fold increase at 4h and 35-fold increase at 12h (IT) after challenge compared to the increase at 1h (ST).

Although meta-analysis could not be performed on TNF-α and IL-6 protein expression over the complete temporal period following one-challenge due to limited studies examining similar time intervals, single studies reported changes in levels of these cytokines [26, 53]. In a human monocytic cell line, TNF-α protein expression reached peak levels at 6h (IT) after LPS challenge or at 12h (IT) after challenge with *L*. *plantarum* genomic DNA, that then slightly decreased from 12h to 48h [53]. TNF-α protein expression pattern in human primary monocytes is also similar to human monocytic cell lines with the maximum increase at 4h (IT), followed by a decline at 24h [26]. Similar to monocytes, human primary macrophages challenged with LPS display elevated TNF-α, with the maximum levels at 4h (IT), its level being constant for 24h, followed by slight reduction at 48h [28]. In mouse primary macrophages, TNF-α protein expression differs from human cells as it reached higher levels at 24h (LT) than at 8h (IT) after challenge with LPS [35]. TNF-α protein levels also reached peak at 24h (LT) after challenge with *L*. *donovani*, and decreased at 48h in a mouse macrophage cell line [13]. However, in mouse bone-marrow dendritic cells, TNF-α protein expression was higher at 8h (IT) than at 24h (LT) after challenge with *H*. *pylori* antigen [105]. These peak increases at 4h, 6h or 12h (IT) of TNF-α protein levels in human monocytes and macrophages correspond to the significant increases at IT identified by the meta-analysis of TNF-α levels in cell lines (S3 Fig in S2 File) and human primary cells (Fig 5). Peak levels of TNF-α protein were delayed to 24h (LT) in mouse macrophages. Zhou *et al*. [35] reported IL-6 and TNF-α protein levels had similar patterns of expression with significant increases at 8h (IT) and 24h (IT) in mouse macrophages when challenged with LPS, and higher levels seen at 24h than 8h. In the murine microglial cell line (BV2), there was a gradual increase of IL-6 protein levels from 1h (ST) to 12h (IT) after LPS challenge [54]. A single study using human gingival fibroblasts, reported a gradual decrease in IL-6 protein level at intervals between 1d (24h –LT) and 7d after LPS challenge [73]. Another study including human fibroblast-like synoviocytes identified a gradual increase in IL-6 protein levels 6h (IT) and 4d interval when challenged with TNF-α [79]. These results indicate that it is difficult to define the temporal periods after one-challenge intervention that are required to reach peak levels of IL-6. Unlike LPS challenge, TLR7 ligand challenge causes peak increases of TNF-α and IL-6 protein expression in mouse cells at 2h (ST) and a decline is observed at 4h [7]. Hence there are differences in time-courses of TNF-α and IL-6 protein expression between cell types (human macrophages versus mouse macrophages, mouse macrophages versus mouse dendritic cells) and types of challenge ligand (e.g. LPS versus TLR7 ligand, LPS versus TNF-α).

#### Effect of IRAK3 expression on inflammatory markers after one-challenge intervention

Gene silencing and knockout approaches are important genetic tools to analyse effects of proteins such as IRAK3 on inflammation and thus outcomes from these approaches are examined in this review. Two studies using human [10] or mouse [13] cell lines reported contradicting effect of IRAK3 on NF-κB reporter gene expression at 6h or 12h (LT_N_) after LPS challenge. In two studies using human cell lines, IRAK3 was reported to have an inhibitory effect on NF-κB DNA binding activity at 6h and 9h (LT_N_) upon LPS stimulation [12, 22]. In contrast, two other studies showed IRAK3 effect increasing NF-κB reporter gene expression in human cell lines at 6h (LT_N_) after IL-1β challenge [8, 10]. A single study, Liu *et al*. [11] showed no significant effect of IRAK3 on NF-κB DNA binding activity at 3h (IT_N_) after challenge with LPS in mouse Kupffer cells (specialized liver macrophages).

**Fig 6:**
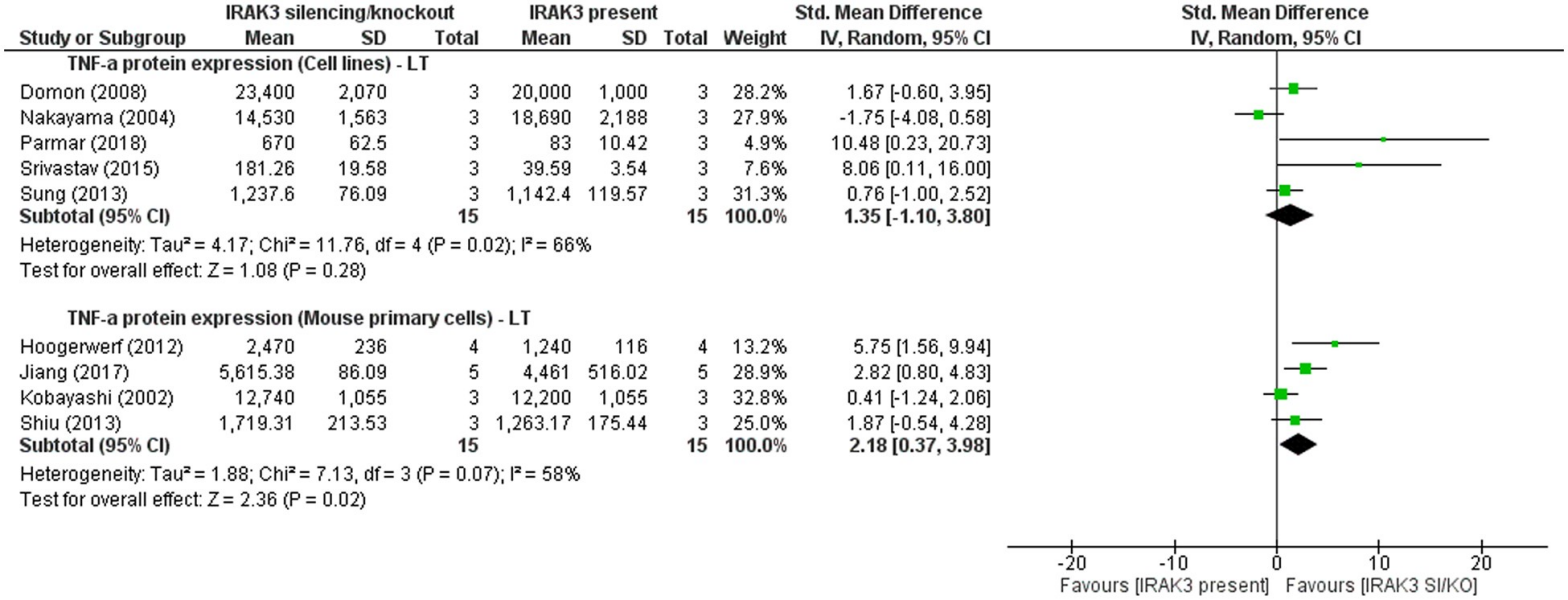
TNF-α protein levels in cell lines and mouse primary cells: IRAK3 silencing/knockout (SI/KO) group versus IRAK3 present group after one-challenge. Intervention was performed using peptidoglycan [21], *L*. *donovani* [13, 67], *H*. *pylori* antigen [105] or LPS [6, 22, 60, 96, 97]. TNF-α protein level was measured at long term (LT; 16h–48h) after one-challenge.

In two studies using mouse cell lines, IRAK3 inhibitory effect on TNF-α protein expression was reported at IT following LPS or *L*. *donovani* [13, 46]. However, meta-analysis indicates no significant effect of IRAK3 on TNF-α protein expression in cell lines at LT after challenge with LPS or peptidoglycan (Fig 6). This differs from mouse primary cell studies, where meta-analysis showed a significant effect of IRAK3 on TNF-α protein levels at LT after challenge with *H*. *pylori* antigen or LPS (Fig 6). In addition, two studies reported opposing findings of IRAK3 effect on TNF-α protein production at IT in mouse primary cells upon one-challenge with *H*. *pylori* antigen or LPS [33, 105]. In a single study, IRAK3 has an effect in decreasing TNF-α protein levels at 24h (LT) in mouse bone marrow-derived macrophages challenged by a TLR7 ligand [7]. Another cell line study shows a significant effect of IRAK3 on reducing TNF-α protein expression at 12h (IT) in macrophage cell lines challenged by titanium particles [65].

Of two included studies using human or mouse cell lines reporting data for IRAK3 effect on IL-6 protein expression at LT after LPS challenge, one study showed inhibitory effect of IRAK3 on IL-6 level, the other also reported a trend of increased IL-6 protein level in IRAK3 silencing cells but no statistical tests were presented [22, 60]. Additionally, three mouse primary cell studies reported IRAK3 effect on IL-6 protein expression at LT after LPS or TLR7 ligand challenge; two studies indicated IRAK3 suppressive effect on IL-6 level [6, 7], whereas the other showed no significant effect [96]. Another study reported IRAK3 has effects on reducing IL-6 protein expression at IT (12h) in mouse bone-marrow derived macrophages challenged by Pam3CSK4 [107].

### Intervention: Two-challenges

In two-challenge interventions (32 of the included 89 studies), cells were challenged first with a TLR or IL-1R ligand and then challenged with the same or a different ligand at a later period. In these 32 studies, the outcomes of interest were measured after durations of 1h–48h following the second challenge. In this section the effect of two-challenges on IRAK3 mRNA and protein expression, NF-κB activity and cytokine protein production are examined by comparing cells receiving one-challenge with cells challenged twice.

#### Effect of two-challenges on IRAK3 mRNA and protein expression

**Fig 7:**
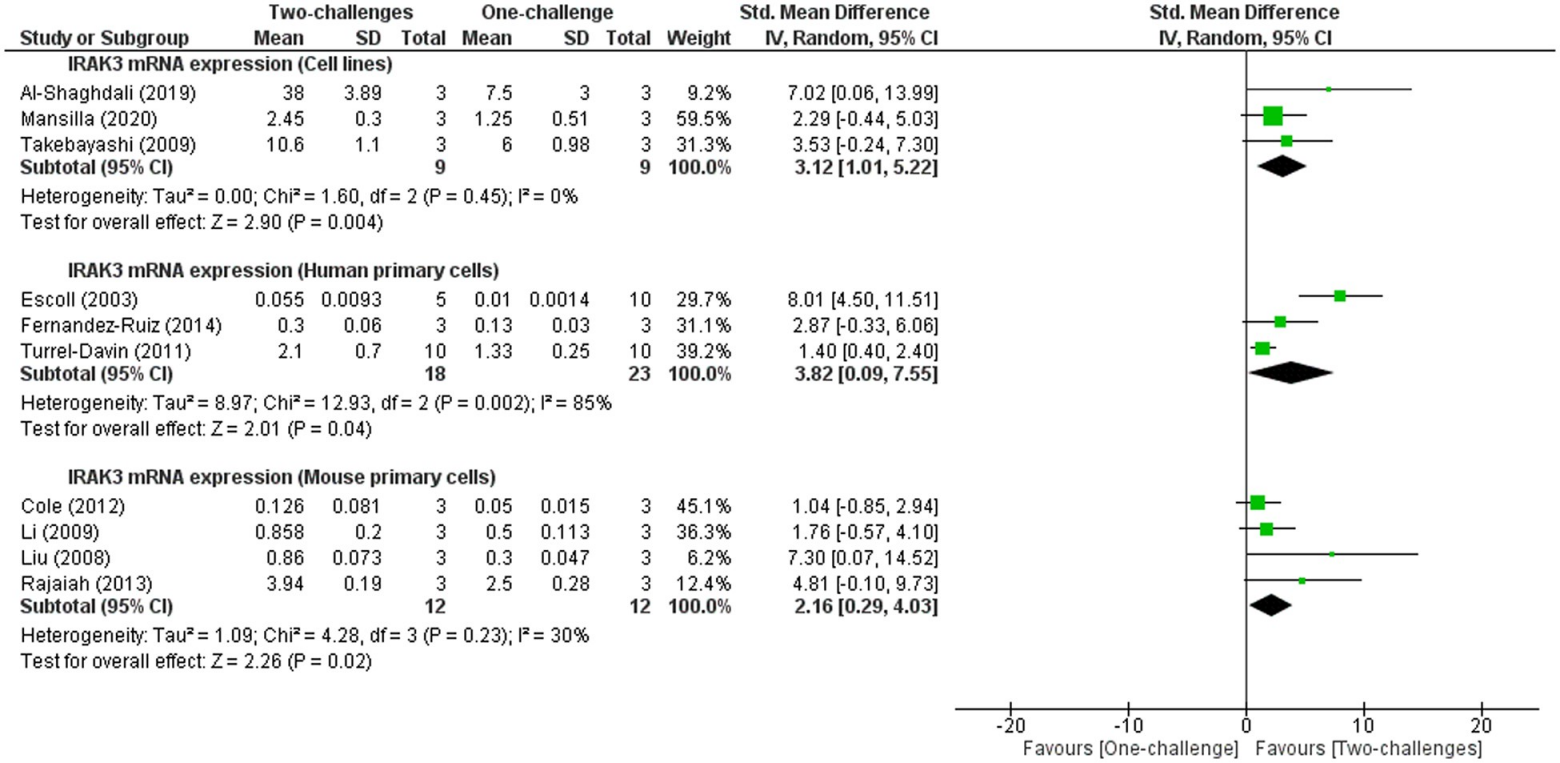
IRAK3 mRNA expression outcomes in cell lines and human or mouse primary cells: One-challenge intervention group versus two-challenge intervention group. In one-challenge intervention group, IRAK3 mRNA expression was measured at 1h [25, 90, 101], 3h [11, 33, 103], 4h [61], 6h [88], 12h [69], 18h [23] after one-challenge with heat-stable enterotoxigenic *E*. *coli* pathogen-associated molecular patterns [69] or LPS. In two-challenge intervention group, cells were first challenged with LPS or *L*. *acidophilus* [69], and then received the second challenge with LPS or heat-stable enterotoxigenic *E*. *coli* pathogen-associated molecular patterns [69]. At an interval after second challenge IRAK3 mRNA expression were measured. The first and second challenge of two-challenge interventions were respectively 4h and 4h [61]; 24h and 1h [25, 101]; 5d and 1h [90]; 15h and 6h [88]; 8h and 3h [33]; 24h and 3h [11]; 24h and 3h [103]; 8h and 24h [80]; 48h and 12h [69].

Meta-analysis of IRAK3 mRNA expression in cell lines, human and mouse primary cells indicates significant increases in IRAK3 mRNA expression measured between 1h and 24h following the second challenge with LPS or heat-stable enterotoxigenic *E*. *coli* pathogen-associated molecular patterns compared to IRAK3 expression between 1h and 18h after one-challenge with LPS or heat-stable enterotoxigenic *E*. *coli* pathogen-associated molecular patterns (Fig 7). Two studies using human primary cells reported significant increase in IRAK3 protein expression at 1h or 24h following the second challenge compared to IRAK3 protein expression at 24h or 1h following one-challenge [80, 87]. Another study reported using immunoblots higher IRAK3 protein expression in monocytic cell lines after two-challenges with LPS and lipoteichoic acid than after one LPS challenge [44]. Another study not included in the meta-analysis represents the only mouse cell study reporting IRAK3 protein expression and showed significantly induced expression of IRAK3 after two-challenges with LPS compared to the level measured at 24h after one LPS challenge in mouse Kupffer cells [11]. An additional study using mouse bone marrow-derived macrophages is excluded from meta-analysis due to differing interventions, and showed higher levels in IRAK3 protein expression in at 5min, 30min, 2h or 24h after second challenge than the levels at 5min, 30min, 2h or 24h after the one-challenge with the same TLR7 ligand [95].

#### Effect of two-challenges on inflammatory markers

Of the included studies, five [11, 44, 52, 77, 78] reported the effects of two-challenges with LPS, TLR7 ligand, TLR2 ligand (Pam3CSK4, lipoteichoic acid) on NF-κB DNA binding activity and NF-κB reporter gene expression. These studies could not be used for meta-analysis, as they differed in cell types used and NF-κB measurement methods. All five studies reported significant decreases in NF-κB DNA binding activity or NF-κB reporter gene expression at 3h and 24h after second challenge when compared to the values from 1h to 24h after one-challenge.

**Fig 8:**
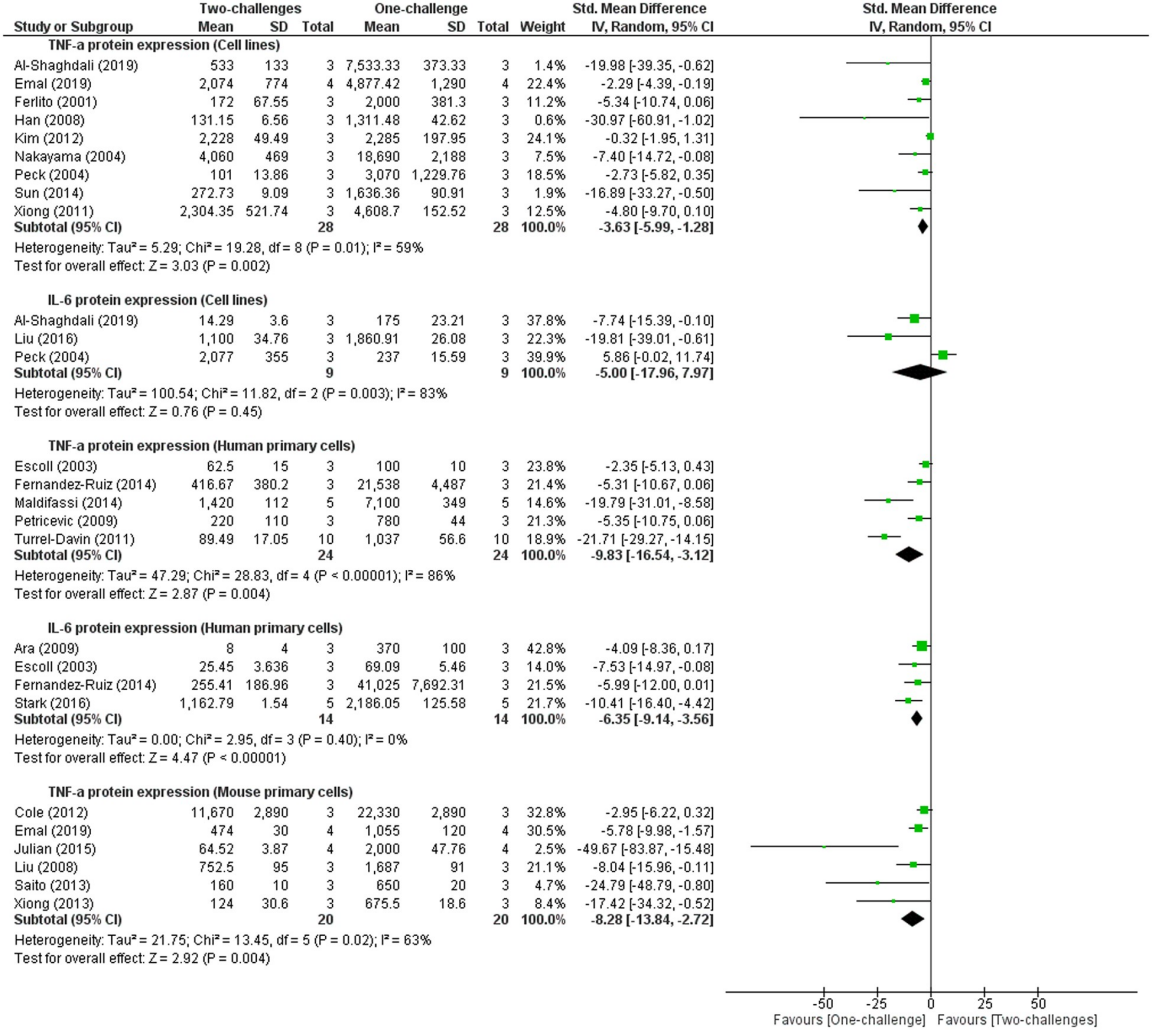
TNF-α and IL-6 protein expression outcome in cell lines, human and mouse primary cells: One-challenge intervention group versus two-challenge intervention group. In one-challenge intervention group, TNF-α and IL-6 protein expression was measured at 3h [11], 4h [44, 47], 6h [53, 87, 88], 8h [33], 12h [54], 18h [23, 49], 24h [21, 25, 59, 66, 73, 80, 83, 90, 98, 104, 111, 113] after one-challenge with peptidoglycan [21] or LPS (other studies included in the meta-analyses). In two-challenge intervention group, cells were first challenged with lipoteichoic acid [44], peptidoglycan [21] or LPS (other studies in the meta-analyses), and then received the second challenge with peptidoglycan [21] or LPS. At an interval after the second challenge, TNF-α and IL-6 protein expression was measured. The first and second challenge of two-challenge interventions were respectively 24h and 18h [23]; 18h and 18h [49]; 18h and 4h [44]; 24h and 6h [53], 16h and 24h [21]; 24h and 24h [25, 59, 66, 73, 83, 98, 104]; 16h and 6h [87]; 20h and 24h [111, 113]; 18h and 12h [54]; 5d and 24h [90]; 4h and 24h [47]; 8h and 24h [80]; 15h and 6h [88]; 8h and 8h [33]; 24h and 3h [11].

Meta-analyses of TNF-α level in studies employing cell lines, human and mouse primary cells indicate marked decreases from 3h to 24h after the second challenge of two-challenge intervention with LPS, peptidoglycan or lipoteichoic acid compared to its levels measured between 3h and 24h after one-challenge with LPS or peptidoglycan (Fig 8). Three studies are not included in the meta-analysis, as they differed in interventions [52, 65], or reported data in inappropriate form for meta-analysis [71]. Adib-Conquy and Cavaillon [71] reported reduced level of TNF-α protein in human monocytes after two LPS challenges compared to one LPS challenge. Zhang *et al*. [65] reported decreased TNF-α protein level in a mouse macrophage cell line (RAW264.7) first challenged by titanium particles followed by a second LPS challenge compared to one-challenge with titanium particles. Similarly, Hassan *et al*. [52] reported decreased level of TNF-α protein in RAW264.7 cells following two-challenges with TLR7 ligand and Pam3CSK4, compared to one-challenge with TLR7 ligand or TLR2 ligand. Meta-analysis of IL-6 protein levels also demonstrates significant reduction in IL-6 protein levels in human primary cells at 1h or 24h after second challenge with LPS compared to the level measured between 16h and 5d after a LPS challenge (Fig 8). Moreover, two mouse primary studies reported significant decrease in IL-6 protein production at 24h or 48h after the second challenge with LPS compared to the level at 24h or 48h following one-challenge [20, 104].

#### Effect of IRAK3 expression on inflammatory markers after two-challenges

A single study reported NF-κB DNA binding activity after two LPS challenges, and this is higher in IRAK3 silenced mouse Kupffer cells than wild type (IRAK3 expressing) cells [11]. Two cell line studies both reported a significant effect of IRAK3 in decreasing TNF-α protein levels following two-challenges with either lipoteichoic acid or peptidoglycan [21, 44]. Another two studies are not suitable for meta-analysis, as they differed in their interventions [52, 65]. Zhang *et al*. [65] reported significant increases in TNF-α protein expression occur in IRAK3 silenced mouse cell lines (RAW264.7) compared to wild type cell lines at 24h after a first titanium particle challenge followed by a second LPS challenge. Similarly, levels of TNF-α protein are higher in IRAK3 silenced mouse cell lines (RAW264.7) than wild type cells at 6h after two-challenges with the first TLR7 ligand challenge and the second Pam3CysSK4 challenge [52]. Two studies using mouse primary cells reported opposing findings for IRAK3 effect on TNF-α protein expression following two-challenges; one study reported significant effect of IRAK3 on reducing TNF-α protein expression [11], whereas other reported no significant effect of IRAK3 [33]. A single study that is not suitable for meta-analysis due to reporting data in inappropriate form for meta-analysis, showed significant effects of IRAK3 on attenuating levels of IL-6 and TNF-α protein expression in mouse bone marrow-derived macrophages upon two LPS challenges [6].

## Discussion

This review provides better understanding of the role of IRAK3 and its upstream and downstream factors in regulating inflammation following one-challenge and two-challenge interventions, and potential involvement in sepsis. The meta-analyses identified IRAK3 mRNA expression increase at ST (5min– 3h) and IT (4h–15h) periods, and IRAK3 protein expression increase at LT (16h–48h) after one-challenge in human primary cells and cell lines (Figs 3 and 4). Levels of secreted pro-inflammatory cytokines (TNF-α and IL-6) were significantly raised at IT and LT intervals after one-challenge (Fig 5 and S3 Fig in S2 File). Comparisons between IRAK3 silencing/knockout and IRAK3 present groups demonstrate an inhibitory effect of IRAK3 on NF-κB DNA binding activity at LT_N_ (6h–24h) in cell lines [12, 22], TNF-α protein expression at IT in cell lines [13, 46], and at LT in mouse primary cells (Fig 6). IRAK3 expression increases more markedly after two-challenges than one-challenge, and this effect is negatively correlated with secreted levels of TNF-α and IL-6 (Figs 7 and 8).

### Intervention: One-challenge

LPS is a prototypical PAMP and was the most widely used receptor ligand in the included studies. The bacterial origin, source and concentration of LPS can influence cell signalling [17, 22, 35, 114]. LPS from *P*. *gingivalis* mainly stimulates cytokine release through the TLR2 pathway, whereas LPS from *E*. *coli* induces cytokine production through TLR4 signalling [115]. Most studies included in this review used LPS from *E*. *coli*, and their findings (Fig 5) correlate with TNF-α and IL-6 protein expression induced by *E*. *coli* LPS at 6h (IT) in a human monocytic cell line rather than the earlier induction by *P*. *gingivalis* LPS at 0.5h (ST) [116]. Bacterial origin of LPS can also affect IRAK3 function, since the effect of IRAK3 silencing on cytokine production was more obvious upon challenge with *P*. *gingivalis* LPS than *E*. *coli* LPS in monocytic cell lines [22]. Moreover, the purity of LPS affects cellular signalling and immune outcomes. *E*. *coli* Ultrapure LPS (Invivogen), which is extracted by successive enzymatic hydrolysis steps and purified by phenol-triethylamine-deoxycholate extraction to remove bacterial lipoproteins that act as TLR2 ligands, specifically activates TLR4, while *E*. *coli* LPS purified simply by ion-exchange chromatography activates both TLR4 and TLR2 [114]. Treatments with *E*. *coli* LPS or *E*. *coli* Ultrapure LPS resulted in differences in time-course and strength of NF-κB dynamic responses [114]. IRAK3 mRNA expression changes were dependent on concentration of the standard LPS [22] or Ultrapure LPS [17]. Sub-nanomolar concentrations of LPS stimulate MEKK3-dependent NF-κB activation induced by IRAK3 over the TAK1 (transforming growth factor β-activated kinase 1)—dependent pathway [35].

IRAK3 mRNA expression is induced from ST, and precedes IRAK3 protein expression at LT after one-challenge in human primary cells and cell lines (Figs 3 and 4). It is noteworthy that significant increases of IRAK3 protein expression (at LT, Fig 4) occur after increases of secreted TNF-α protein levels (at IT and LT, Fig 5 and S3 Fig in S2 File). Nitric oxide is elevated during bacterial infection and in plasma concentration of patients with septic shock [117]. It increased TNF-α mRNA expression at 2h (ST) in human monocytes, which in turn induced IRAK3 mRNA and protein expression at 8h (IT) and 20h (LT), respectively [75]. A blocking antibody against TNF-α also suppressed IRAK3 expression induced by nitric oxide, further indicating TNF-α–dependent effects on IRAK3 expression [75]. Therefore, it appears that elevated nitric oxide levels produced in response to bacteria or endotoxin result in significant increases of TNF-α that precede and contribute to induction of IRAK3 expression. Nitric oxide inhibits the molecular interaction of IRAK1 and TRAF6 and down-regulates inflammatory responses by stabilising the NF-κB inhibitor, alpha (IκBα) [75].

The temporal patterns of cytokine expression appear to be affected by cell sources (e.g. human cells versus mouse cells). TNF-α protein expression reaches peak levels at IT in human primary cells and human cell lines, but at LT in mouse primary cells [13, 26, 28, 35, 53]. Moreover, the maximum level of TNF-α protein expression in human cell lines was observed at IT, and in mouse cell lines at LT, indicating TNF-α expression patterns of human and mouse cell lines are similar to human and mouse primary cells respectively [13, 26, 35, 53]. In spite of differences in the time interval to reach peak levels, significant increases of TNF-α and IL-6 protein levels at IT and LT upon one-challenge were reported in both human and mouse primary cells or cell lines (Fig 5 and S3 Fig in S2 File); and IL-6 levels and NF-κB activity also significantly increased at ST and LT_N_ respectively, in both human and mouse cell lines (S2 Fig in S2 File) [48, 54]. Whereas human and mouse primary cells showed dissimilarity in IRAK3 mRNA expression after one-challenge (Fig 3 and S1 Fig in S2 File), IRAK3 mRNA expression patterns in human and mouse cell lines following one- or two-challenges have high similarity (Figs 3 and 7). However, IRAK3 protein expression markedly increased (1.5-fold) at ST after one-challenge in mouse cell line [13], but not in human cell lines [22, 50]. The results indicate differences between TNF-α cytokine production and IRAK3 mRNA or protein expression in humans and mice at least in the temporal framework of this analysis. This is relevant, even though the gene expression patterns of the mouse model closely mimics those of human conditions [118], there is concern that differences in cellular composition between mouse models and human may contribute to failures in reflecting the human immune system [42].

One-challenge intervention model mimics the initial hyper-inflammatory phase of sepsis. The hyper-inflammatory phase presents with dramatic increases of various potent cytokines (called a cytokine storm) including TNF-α and IL-6 in patients with sepsis, and in *in vivo* mice and human studies [41, 119]. IL-6 protein level is significantly elevated during acute phase of sepsis (in 4d since sepsis diagnosis) in critically ill patients compared to non-critically ill and healthy ones [120]. TNF-α level was significantly reduced in non-survivors of elderly patients with sepsis on the first day of diagnosis compared to survivors [121]. Moreover, both TNF-α and IL-6 levels of septic serum samples were highly increased, and could be used in diagnosis and therapeutic management of neonatal sepsis [122]. *In vitro* cell models also demonstrated cytokine storm upon endotoxin stimulation, as confirmed for TNF-α and IL-6 at IT and LT in this review (Fig 5 and S3 Fig in S2 File). A human *in vivo* model in which healthy volunteers were injected with LPS demonstrated increases of IRAK3 mRNA and protein expression at IT, and peak induction of TNF-α protein at 1.5h (ST) [17]. Another *in vivo* study where human subjects were administered LPS for five consecutive days reported the peak level of TNF-α at 1.5h (ST) on day 1 followed by peak level of IL-6 at 2h–3h (ST) [123]. Mice *in vivo* models injected with LPS also show significant increases in IRAK3 mRNA levels from ST to LT and protein levels at LT [16, 61, 81, 113, 124, 125], with peak increases of TNF-α protein production at ST [108, 126] and at LT [44, 127]. Thus, the induction of IRAK3 mRNA and protein expression, TNF-α and IL-6 protein level appeared to occur sooner in *in vivo* models than *in vitro* cell models. Differences in inflammatory responses between *in vitro* and *in vivo* models can be due to the involvement of other regulators such as glucocorticoids or neuroprotective peptides present *in vivo* model [123].

The effect of IRAK3 on NF-κB activity differs when challenged with IL-1β or LPS [8, 10, 12, 22], suggesting that different ligands activate alternative signalling pathways involving IRAK3. This idea is further reinforced by distinctive functions of IRAK3 upon IL-33 challenge in dendritic cells where it stimulates the expression of inflammatory cytokines including IL-6 [9]. Following IL-33 stimulation, IRAK3 is bound to and isomerised by transcription factors peptidylprolyl cis/trans isomerase, NIMA-interacting 1 (PIN1) to release from myddosome complex and translocate into nucleus to induce expression of selected pro-inflammatory cytokines (IL-6, IL-5 and IL-13) in dendritic cells [9]. IRAK3 gene silencing or knockout studies show IRAK3 has subtle effects suppressing temporal expression of TNF-α and IL-6 [6, 7, 13, 22, 46] (Fig 6). Most studies modulating IRAK3 expression using cell lines or human primary cells relied on gene silencing technique. Short interfering RNAs (siRNAs) that are oligonucleotide sequences targeting several exon regions of IRAK3 mRNA were transfected to enhance the degradation of IRAK3 mRNA or transcripts, leading to decreased post-transcriptional protein expression of IRAK3 [13, 21, 22, 44, 46, 60, 80, 67]. These studies also showed evidence of valid knockdown of IRAK3 protein expression on immunoblots or RT-PCR (Real-time polymerase chain reaction). The knockout efficiency of gene silencing can be variable and is unable to achieve 100%, whereas gene knockout manipulation can obtain 100% deletion via repressing gene transcription of IRAK3 or generating IRAK3 frame shift DNA mutation [128]. Thus, gene silencing may not reflect the complete loss-of-function of IRAK3 on inflammation, compared to IRAK3 gene knockout [128]. TNF-α protein expression in cell lines at LT following one-challenge appeared to be affected by IRAK3 silencing to different extents compared to gene knockout cells (Fig 6). These findings are based on different cell types (gene silencing used in cell lines versus mouse primary cells from IRAK3 knockout mice) which can also be the main cause for this dissimilarity in IRAK3 temporal effects. Moreover, the incomplete deletion of gene silencing offers less pronounced phenotypes than gene knockout, which can be beneficial for cell fitness and viability [128]. However, using siRNA for gene silencing can induce off-target effects because of the possible interaction of siRNA with some regions of the mRNA 3’-UTR (untranslated region), or because the introduction of exogenous siRNA into a cell line can change or displace the cell-specific system of endogenous microRNAs [128]. The off-target phenotypes may rule over the on-target phenotypes caused by silencing of the gene of interest [128]. On the other hand, IRAK3 knockout manipulations have been widely performed in mouse primary cell studies using gene targeting with homologous recombination in embryonic stem cells, and the target knockout in the mouse IRAK3 sequence should be considered. Nevertheless, gene targeting with homologous recombination also has two major disadvantages. Firstly, there might be no observable phenotypical effects caused by full silencing of the target gene due to the compensation by another gene family encoding a protein with highly similar amino acid sequence [129]. The second problem is genetic linkage which makes difficulty to identify the phenotypical differences caused by the knockout mutation or the genetic difference in the regions flanking the target locus following animal cross-breeding [129]. Notably, an issue related to generating IRAK3 knockout mice via gene targeting with homologous recombination has been raised [130]. IRAK3 has 12 exons; the targeted deletion of exons 9–11 belonged to kinase domain by homologous recombination inserting neomycin resistance cassette was firstly performed by Kobayashi *et al*. [6] and then used in most of included studies [6, 33, 96, 105]; only one included study targeted deletion of exon 3 for IRAK3 knockout in mouse [97]. However, in at least one instance, loss of neomycin cassette took place in targeted exons 9–11 deletion at mRNA level, and a splicing event occurred to join exon 8 and exon 12 and generate a splice variant of IRAK3 in bone-marrow derived macrophages following challenges [130]. It is also noted this truncated protein is more potent at inducing NF-κB activity than IRAK3 wildtype [130]. Therefore, although the inhibitory effect of IRAK3 on inflammation was also reported in IRAK3 silencing experiments and confirmed in meta-analyses (Fig 6), the splice variant is possibly created following IRAK3 knockout and may affect the reliance of functional studies using IRAK3 knockout models. To ensure the comprehensiveness of the systematic review, data from all published studies performing IRAK3 gene silencing or knockout techniques are included in meta-analyses.

Glucocorticoids are potent anti-inflammatory agents with evidence for beneficial effects in severe sepsis treatment. Long-course administration of low-doses of corticosteroid reduced mortality of patients with sepsis and the length of stay in the intensive care unit [131, 132]. One of anti-inflammatory actions of glucocorticoids is via suppression of degradation of IRAK1 and IRAK4, and inhibition of TRAF6 activation at 30min after LPS challenge in microglial cell lines [133]. In addition, glucocorticoids bind to the intracellular glucocorticoid receptor, which in turn migrates to the nucleus to bind to glucocorticoid response elements in promoters of several genes including IRAK3 [81]. Glucocorticoids augment IRAK3 expression at mRNA and protein levels in response to *H*. *influenzae* infection in cell lines and mouse primary cells, thereby decreasing *H*. *influenzae*–induced expression of inflammatory cytokines including TNF-α and IL-6 at 24h (LT) in wildtype mice, but this suppressive effect is disabled in IRAK3 knockout mice [81]. Another transcription factor, hypoxia-inducible factor-1α (HIF-1α) is induced by Gram-negative endotoxin challenge in mice and upregulated in sepsis-monocytes [134]. Treatment of human monocytes with activators of HIF-1α upregulate IRAK3 mRNA and protein expression between 0.5h (ST) to 24h (LT) and also reduce endotoxin-induced TNF-α and IL-6 expression at IT and LT, which does not occur in IRAK3 silenced cells [134]. Thus, both the glucocorticoid receptor and HIF-1α inhibit production of inflammatory cytokines through enhancement of IRAK3 expression [81, 134].

### Intervention: Two-challenges

This review indicates significant increases in IRAK3 mRNA and protein expression, and reductions in TNF-α and IL-6 protein level between 1h and 24h following the second challenge (Figs 7 and 8). Thus, there is a strong negative correlation between IRAK3 mRNA and protein expression and inflammatory cytokine TNF-α and IL-6 expression after two-challenge intervention. This negative correlation reflects the context of endotoxin tolerance in which IRAK3 expression is increased significantly to limit inflammatory cytokine levels in response to repeated endotoxin challenges [6, 16, 17, 89]. Various endotoxin tolerant examples including septic patients’ samples *ex vivo* treated with endotoxin and *in vivo* mouse or human models similarly reported profound reduction of TNF-α and IL-6 [19, 119, 123]. For instance, an *in vivo* human study performing LPS administration for five consecutive days showed a significant attenuation of TNF-α and IL-6 on the fifth compared to levels on the first day [123]. The function of IRAK3 on endotoxin tolerance is further confirmed by the higher TNF-α level in IRAK3 silencing or knockout—monocytes, macrophages or Kupffer cells challenged twice than wild type cells [6, 11, 21, 44]. However, IRAK3 functions in endotoxin tolerance can be influenced by cell types, since IRAK3 does not inhibit TNF-α level in mouse bone marrow dendritic cells, but regulates the expression of cell surface molecules such as CD80 (cluster of differentiation 80) instead [33].

Identifying the time frames of these events in different cell models is relevant, as IRAK3 is involved in sepsis pathophysiology [14, 135]. The dysregulated cytokine production responses of the first phase of sepsis that can cause organ injury or dysfunction are followed by immunosuppression characterized by irresponsiveness to repeated infections with pathogens [18]. Notably, IRAK3 plays a role in regulating immunosuppression in sepsis. IRAK3 mRNA expression is up-regulated in blood samples of patients with sepsis compared with healthy controls [14]. Clinical samples of sepsis patients show increased IRAK3 mRNA levels and significant decreases in TNF-α and IL-6 protein levels at 4h (IT) after *ex vivo* LPS challenge [89] or decreased TNF-α protein levels at IT or LT [88], whereas samples from healthy controls demonstrate the hyper-inflammatory phase of sepsis. The meta-analyses of TNF-α and IL-6 protein levels after two-challenges show similarities to the clinical sepsis samples (Fig 8). However, Dong *et al*. [135] reported higher levels of TNF-α and IL-6 proteins in clinical samples of sepsis patients at 4h (IT) after *ex vivo* LPS challenge than in samples of healthy controls, which reflects a hyper-inflammatory response phase of sepsis similar to the meta-analysis of one-challenge (Fig 5). Potentially, variations in TNF-α and IL-6 protein expression in samples from sepsis patients after LPS challenge may be explained by when blood samples were taken in relation to the different phases of sepsis. *In vitro* models of human cell lines and primary cell cultures at 4h (IT) after one-challenge or two-challenges with LPS or bacteria showed significant effects of interventions on IRAK3, TNF-α and IL-6 protein expression which are comparable to outcomes reflecting the two phases of sepsis in patients. Thus, *in vitro* cell culture models challenged with bacteria or LPS can mimic sepsis conditions and are useful to study the molecular functions of IRAK3 in endotoxin tolerance and sepsis. However, blood samples from sepsis patients need to be tested for IRAK3, TNF-α and IL-6 protein levels at more time intervals after endotoxin challenge to clarify whether the patterns of temporal expression of these molecules reflect *in vitro* human cell culture models. Such studies also need to consider the phase at which the blood is collected from the sepsis patients.

## Strengths and limitations

To our knowledge, no review involving meta-analyses aimed at elucidating how IRAK3 impacts inflammation through modulation of inflammatory markers has been conducted to date. The systematic search conducted reduced the likelihood of missing relevant studies. Meta-analyses are carried out to consolidate varied results from different studies. The consistency of meta-analyses can be influenced by the large values of heterogeneity across the included studies. While low levels of heterogeneity were observed between cell line studies, higher levels of heterogeneity were observed across both mouse and human primary cell studies. These high levels of heterogeneity could be influenced by diverse behaviours of different cell sources or donors, whereas cell lines are stably-established. Where high levels of heterogeneity occurred between studies, this was considered while interpreting results. Several studies from the final library could not be included in meta-analyses; for instance, they differed in intervention chemicals, or the format of reported data was inappropriate for meta-analysis. In order to perform meta-analyses for each outcome, data must be in the similar measurement unit and format (e.g., direct measured value or fold-change), and arranged in appropriate comparison groups (e.g. similar stimulants, signal pathways, cell types). Moreover, study data should indicate mean values, precise number of samples (n), and standard deviation or standard error. Another limitation arises from the number studies employing specific techniques on the cell types examined identified in this review. For example, only one study used human primary cells in which IRAK3 was silenced [122].

## Conclusions

**Fig 9:**
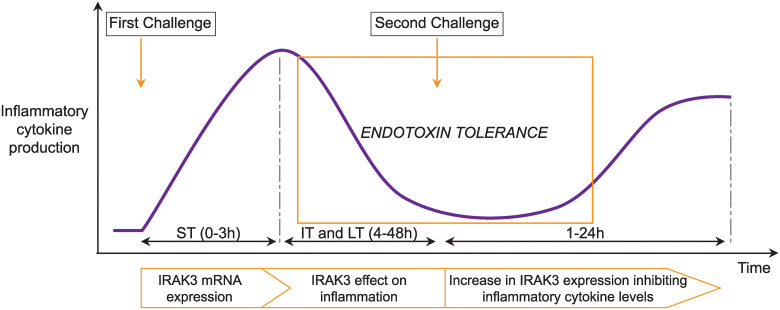
Scheme of the temporal expression and effect of IRAK3 on inflammation after the first and second challenges in cell models. After the first challenge, IRAK3 mRNA expression increases at ST (0h–3h), and as IRAK3 protein is expressed, inhibitory effects on inflammation occur at IT and LT (4h–48h). Following the second challenge, greater increases in IRAK3 mRNA and protein expression occur between 1h and 24h compared to the first challenge, suppressing inflammatory signalling.

This systematic review and meta-analyses reveals temporal patterns of IRAK3 mRNA and protein expression, NF-κB activity and TNF-α and IL-6 protein expression following one- or two-challenge interventions in cell lines, human and mouse primary cells (Fig 9). In cell lines, NF-κB activity is increased between 6h and 24h, and induced where IRAK3 is silenced upon LPS challenge. In human monocytes and macrophages challenged by bacteria or bacterial components, TNF-α protein expression reaches peak levels at intervals between 4h and 12h (IT), followed by slight decreases at 24h and 48h (LT). However, the pattern of TNF-α protein expression in mouse macrophages differs from human cells, with a delayed peak at 24h (LT) followed by decreases at 48h (LT). IL-6 protein expression is similar in cell lines and mouse primary cells. IRAK3 effect limits TNF-α peaks at IT in cell lines and LT in mouse primary cells. Due to a lack of sufficient data to carry out meta-analysis, temporal effect of IRAK3 on IL-6 protein level remains inconclusive. Following a second challenge, increased levels of IRAK3 expression remain steady and correlate with decreased levels of inflammatory cytokines at 1h and until at least 24h. The inhibitory impact of IRAK3 on TNF-α expression after two-challenges is consistently confirmed in cell line studies. In summary, this review highlights differences between human and mouse cells in terms of temporal actions of IRAK3 and cytokine protein expression patterns. This information is useful in designing experiments to explore actions of IRAK3 at the molecular level and how it influences expression of inflammatory cytokines using cell lines to model sepsis and endotoxin tolerance, with respect to different intervention chemicals and time post-treatment. A future systematic review and meta-analyses on *in vivo* studies may lead to further understanding of IRAK3 action on inflammation and identify possible differences between *in vitro* and *in vivo* models.

## Supporting information

S1 Checklist PRISMA 2009 checklist. (DOC)

S1 File Supplementary tables. (PDF)

S2 File Supplementary figures and references. (PDF)
