# Supplementary material for: Analysis of interleukin-1 receptor associated kinase-3 (IRAK3) function in modulating expression of inflammatory markers in cell culture models: A systematic review and meta-analysis
Source: PLoS One. 2020 Dec 31;15(12):e0244570. doi: 10.1371/journal.pone.0244570 (PMC7774834; doi:10.1371/journal.pone.0244570)
Supplement: S1 File — (PDF) [file pone.0244570.s002.pdf]

SUPPLEMENTARY TABLES

S1 Table. Description of intervention chemicals used in included studies of this review.

**Intervention chemicals**	**Description**
Lipopolysaccharide (LPS)	A component of outer membrane of Gram-negative bacteria.
Pam3CysSerLys4 (Pam3CSK4)	A synthetic triacylated lipopeptide of bacteria.
Lipoteichoic acid (LTA)	A constituent of cell wall of Gram-positive bacteria.
Peptidoglycan	A polymer forming the layer outside plasma membrane of most bacteria.
S-nitrosoglutathione (GSNO)	A donor of nitric oxide.
Transforming growth factor beta (TGF-β), Interleukin-13 (IL-13)	Anti-inflammatory cytokines.
Granulocyte-macrophage colony-stimulating factor (GM-CSF), IL-1, IL-1β, Tumour necrosis factor alpha (TNF-α), Interferon gamma (IFN-ϒ)	Inflammatory cytokines.
Paramethoxyamphetamine	An activator of protein kinase C.
6-methylprednisolone	A glucocorticoid.
Surfactant protein A	Lung collectin.
Titanium and bone cement particles	Used in *in vitro* experiments to mimic implant-derived debris in joint arthroplasty.
Chemical names: R837, R848, 1V136	Toll-like receptor 7 (TLR7) agonist.
Hyperoxia	Cells cultured in 95% oxygen instead of room air.

S2 Table. Characteristics of included studies.

No	Study	Cell lines/types and number	Stimulant	Intervention duration	Comparison groups	Outcome measure of interest	Effect sizes
1	Adib-Conquy and Cavaillon *et al*. (2002)	Human peripheral blood mononuclear cells (PBMCs)	IFN-ϒ or GM-CSF alone or simultaneously with 1 or 100 ng/ml *Escherichia coli* (0111:B4) LPS ;<br>100 ng/ml LPS	24h (1st), 24h (2nd)	Group 1: One-challenge<br>Group 2: Control	TNF-α protein expression	Not estimable.
2	Al Mubarak *et al*. (2018)	Human tracheobronchial epithelial cells and alveolar macrophages<br>(n = 4)	10 ng/ml LPS (not mentioned bacterial origin)	4h<br><br>24h<br><br>4h<br><br>24h	Group 1: One-challenge<br>Group 2: Control<br><br>Group 1: One-challenge<br>Group 2: Control	IRAK3 mRNA expression<br><br>TNF-α protein expression	(Positive favours One-challenge)<br>IRAK3 mRNA expression: 0.18 (-0.81, 1.16)<br>IRAK3 mRNA expression: 1.52 (0.37, 2.67)<br><br>(Positive favours One-challenge)<br>TNF-α protein expression: 0.71 (-0.76, 2.18)<br>TNF-α protein expression: 0.83 (-0.67, 2.33)
3	Almeida *et al*. (2009)	Human PBMCs and monocytes	*Mycobacterium tuberculosis*	24h	Group 1: One-challenge<br>Group 2: Control	TNF-α protein expression	Not estimable.
4	Al-Shaghdali *et al*. (2019)	THP-1 (human monocyte) - derived macrophages<br>(n = 3)	100 ng/ml *Escherichia coli* K12 LPS<br><br>100 ng/ml LPS	24h<br><br>24h (1st), 18h (2nd)	Group 1: One-challenge<br>Group 2: Control<br><br>Group 1: Two-challenges<br>Group 2: One-challenge	TNF-α protein expression<br><br>IRAK3 mRNA expression<br><br>IL-6 protein expression<br><br>TNF-α protein expression<br>IL-6 protein expression<br><br>IRAK3 mRNA expression	(Positive favours One-challenge)<br>TNF-α protein expression: 23.08 (0.74, 45.42)<br><br>(Positive favours One-challenge)<br>IRAK3 mRNA expression: 9.13 (0.17, 18.09)<br><br>(Positive favours One-challenge)<br>IL-6 protein expression: 8.22 (0.12, 16.31)<br><br>(Negative favours One-challenges)<br>TNF-α protein expression: -19.98 (-39.35, -0.62)<br>(Negative favours One-challenges)<br>IL-6 protein expression: -7.74 (-15.39, -0.10)<br>(Positive favours Two-challenges)<br>IRAK3 mRNA expression: 7.02 (0.06, 13.99)

5	Ara *et al*. (2009)	Human peripheral blood monocytes<br>(n = 3)	10 ng/mL *Porphyromonas gingivalis* 381 and *Escherichia coli* O128:B12 LPS	24h<br><br>24h (1st), 24h (2nd)	Group 1: One-challenge<br>Group 2: Control<br><br>Group 1: Two-challenges<br>Group 2: One-challenge	IL-6 protein expression<br><br>IL-6 protein expression	(Positive favours One-challenge)<br>IL-6 protein expression: 2.33 (1.14, 3.52)<br><br>(Negative favours One-challenges)<br>IL-6 protein expression: -4.09 (-8.36, 0.17)
6	Brudecki *et al*. (2013)	THP-1 (human monocyte)<br>(n = 3)	1 µg/ml *Escherichia coli* O111:B4 LPS	4h<br><br>2h	Group 1: One-challenge.<br>Group 2: Control<br><br>Group 1: One-challenge<br>Group 2: Control	TNF-α protein expression<br>IL-6 protein expression<br><br>TNF-α protein expression<br>IL-6 protein expression	(Positive favours One-challenge)<br>TNF-α protein expression: 9.44 (0.19, 18.69)<br>(Positive favours One-challenge)<br>IL-6 protein expression: 16.53 (0.60, 38.37)<br><br>Not estimable<br><br>(Negative favours One-challenge)<br>IL-6 protein expression: 1.60 (-0.63, 3.82)
7	Chen *et al*. (2007)	Human PBMCs<br>(n = 22)	1 µg/ml highly purified LPS from *Escherichia coli* K235	6h	Group 1: One-challenge<br>Group 2: Control	TNF-α protein expression	(Positive favours One-challenge)<br>TNF-α protein expression: 6.26 (4.77, 7.76)
8	Chen *et al*. (2012)	Cardiac fibroblasts isolated from normal mouse hearts<br>(n = 7)	LPS (Sigma Aldrich) (conc. not shown and not mentioned bacterial origin)	4h	Group 1: One-challenge<br>Group 2: Control	IRAK3 mRNA expression	(Positive favours One-challenge)<br>IRAK3 mRNA expression: 10.44 (5.76, 15.12)
9	Cole *et al*. (2012)	Mouse bone marrow dendritic cells (BMDCs)<br>(n = 3)	10 ng/ml *Escherichia coli* O127:B8 LPS<br><br>10 ng/ml LPS<br><br>10 ng/ml LPS, 100 ng/ml LPS	3h<br><br>8h<br><br>8h (1st), 3h (2nd)	Group 1: One-challenge<br>Group 2: Control<br><br>Group 1: One-challenge<br>Group 2: Control<br><br>Group 1: Two-challenges	IRAK3 mRNA expression<br><br>IRAK3 mRNA expression<br><br>IRAK3 mRNA expression	(Positive favours One-challenge)<br>IRAK3 mRNA expression: 2.53 (-0.39, 5.45)<br><br>(Positive favours One-challenge)<br>IRAK3 mRNA expression: 5.85 (-0.02, 11.72)<br><br>(Positive favours Two-challenges)<br>IRAK3 mRNA expression: 1.04 (-0.85, 2.94)

					Group 2: One-challenge		
			10 ng/ml LPS	8h	Group 1: One-challenge<br>Group 2: Control	TNF-α protein expression	(Positive favours One-challenge)<br>TNF-α protein expression: 8.53 (0.14, 16.91)
			10 ng/ml LPS, 100 ng/mL LPS	8 h (1st), 8h (2nd)	Group 1: Two-challenges<br>Group 2: One-challenge	TNF-α protein expression	(Negative favours One-challenge)<br>TNF-α protein expression: -2.95 (-6.22, 0.32)
			10 ng/ml LPS	8h	Group 1: IRAK3 silencing/knockout<br>Group 2: IRAK3 present	TNF-α protein expression	Not estimable.
			10 ng/ml LPS, 100 ng/ml LPS	8 h (1st), 8h (2nd)	Group 1: IRAK3 silencing/knockout<br>Group 2: IRAK3 present	TNF-α protein expression	Not estimable.
10	Daskalaki *et al*. (2019)	RAW 264.7 (mouse macrophage)<br>(n = 3)	100 ng/ml *Escherichia coli* O111:B4 LPS	24h	Group 1: One-challenge<br>Group 2: Control	IRAK3 mRNA expression<br><br>TNF-α protein expression	(Positive favours One-challenge)<br>IRAK3 mRNA expression: 5.05 (-0.08, 10.18)<br>(Positive favours One-challenge)<br>TNF-α protein expression: 11.42 (0.28, 22.57)
11	Del Fresno *et al*. (2005)	Human monocytes<br>(n = 5)	10 ng/ml *Salmonella abortus* LPS	3h	Group 1: One-challenge<br>Group 2: Control	IRAK3 mRNA expression	(Positive favours One-challenge)<br>IRAK3 mRNA expression: 3.01 (0.91, 5.11)
				6h	Group 1: One-challenge<br>Group 2: Control	IRAK3 mRNA expression	(Positive favours One-challenge)<br>IRAK3 mRNA expression: 13.21 (5.67, 20.76)
				24h	Group 1: One-challenge<br>Group 2: Control	IRAK3 mRNA expression	(Positive favours One-challenge)<br>IRAK3 mRNA expression: 2.41 (0.57, 4.25)

12	Del Fresno *et al*. (2007)	Human CD14+ monocytes<br>(n = 10)	10 ng/ml *Salmonella abortus* LPS	1h<br><br><br>24h	Group 1: One-challenge<br>Group 2: Control	IRAK3 mRNA expression<br><br>IRAK3 protein expression<br><br>TNF-α protein expression<br><br><br>IL-6 protein expression	(Positive favours One-challenge)<br>IRAK3 mRNA expression: 0.29 (-0.59, 1.17)<br><br>(Positive favours One-challenge)<br>IRAK3 protein expression: 2.33 (1.14, 3.52)<br><br>(Positive favours One-challenge)<br>TNF-α protein expression: 26.94 (17.58, 36.30)<br><br>(Positive favours One-challenge)<br>IL-6 protein expression: 63.19 (41.32, 85.07)
13	Domon *et al*. (2008)	THP-1 (human monocyte)<br>(n = 3)	1 µg/ml LPS from *Porphyromonas gingivalis* and *Escherichia coli*<br><br><br><br><br>0.1 or 1 µg/ml *Porphyromonas gingivalis* and *Escherichia coli* LPS<br>1 µg/ml LPS from *Escherichia coli*<br><br><br><br>1 µg/ml LPS from *Escherichia coli*	9h<br><br><br><br><br><br><br><br>1h – 3h – 6h – 9h – 12h<br><br><br><br>24h<br><br><br><br><br>24h	Group 1: One-challenge<br>Group 2: Control<br>Group 1: IRAK3 silencing/knockout group.<br>Group 2: IRAK3 present group.<br>Group 1: One-challenge<br>Group 2: Control<br><br><br>Group 1: One-challenge<br>Group 2: Control<br><br><br>Group 1: IRAK3 silencing/knockout group<br>Group 2: IRAK3 present group	NF-κB p65 activation<br><br><br><br><br><br><br><br>mRNA and protein expressions of IRAK3<br><br><br><br>TNF-α protein expression<br>IL-6 protein expression<br><br>TNF-α protein expression<br>IL-6 protein expression	Not estimable<br><br><br>Not estimable<br><br><br><br><br>Not estimable<br><br><br><br><br>(Positive favours One-challenge)<br>TNF-α protein expression: 21.49 (0.68, 42.31)<br>IL-6 protein expression: 5.65 (-0.04, 11.33)<br><br><br>(Positive favours IRAK3 silencing/knockout)<br>TNF-α protein expression: 1.67 (-0.60, 3.95)<br>Not estimable.
14	Du *et al*. (2014)	293T (human embryonic kidney epithelial	2 ng/ml IL-1β	6h	Group 1: IRAK3 silencing/knockout group	NF-κB activation	(Negative favours IRAK3 present)<br>NF-κB activation: -4.61 (-0.87, -1.15)

		cell) transfected with IRAK3 and control vector (n = 4)	1 ng/ml Ultrapure LPS (Invivogen)		Group 2: IRAK3 present group<br>Group 1: One-challenge<br>Group 2: Control	TNF-α protein expression<br>IL-6 protein expression	(Positive favours One-challenge)<br>TNF-α protein expression: 81.56 (25.44, 137.67)<br>IL-6 protein expression: 11.81 (3.57, 20.06).
15	Emal et al. (2019)	Mouse bone marrow macrophages (n = 4)	100 µg/ml LPS from *Escherichia coli*, 100 ng/ml LPS from *Escherichia coli*	24h (1st), 4h (2nd)	Group 1: Two-challenges<br>Group 2: One-challenge	TNF-α protein expression	(Negative favours One-challenge)<br>TNF-α protein expression: -5.78 (-9.98, -1.57)
			100 µg/ml LPS from *Escherichia coli*	4h	Group 1: One-challenge<br>Group 2: Control	IRAK3 mRNA expression	(Positive favours One-challenge)<br>IRAK3 mRNA expression: 13.20 (4.02, 22.38)
		RAW264.7 (n = 4)	100 µg/ml LPS from *Escherichia coli*, 100 ng/ml LPS from *Escherichia coli*	24h (1st), 4h (2nd)	Group 1: Two-challenges<br>Group 2: One-challenge	TNF-α protein expression	(Negative favours One-challenge)<br>TNF-α protein expression: -2.29 (-4.39, -0.19)
			100 µg/ml LPS from *Escherichia coli*	4h	Group 1: One-challenge<br>Group 2: Control	IRAK3 mRNA expression	(Positive favours One-challenge)<br>IRAK3 mRNA expression: 18.15 (5.59, 30.71)
		Human whole blood cells (n = 4)	500 ng/ml LPS from *Escherichia coli*	4h	Group 1: One-challenge<br>Group 2: Control	TNF-α protein expression	(Positive favours One-challenge)<br>TNF-α protein expression: 1.92 (0.27, 3.56)
16	Escoll *et al*. (2003)	Human monocytes (n = 10)	100 ng/ml *Salmonella abortus* LPS	6h	Group 1: One-challenge<br>Group 2: Control	IRAK3 mRNA expression	(Positive favours One-challenge)<br>IRAK3 mRNA expression: 3.98 (2.35, 5.62)
			100 ng/ml LPS	24h			(Positive favours One-challenge)<br>IRAK3 mRNA expression: 3.42 (1.95, 4.89)
		(n = 10 – One-challenge; n = 5 – Two-challenges)	10 ng/ml LPS, 100 ng/ml LPS	24h (1st), 1h (2nd)	Group 1: Two-challenges<br>Group 2: One-challenge		(Positive favours Two-challenges)<br>IRAK3 mRNA expression: 8.01 (4.50, 11.51)

			10 ng/ml LPS<br><br><br><br><br><br>10 ng/ml LPS, 100 ng/ml LPS	24h<br><br><br><br><br><br>24h (1st), 24h (2nd)	Group 1: One-challenge<br>Group 2: Control<br><br><br><br>Group 1: Two-challenges<br>Group 2: One-challenge	TNF-α protein expression<br><br>IL-6 protein expression<br><br>TNF-α protein expression<br><br>IL-6 protein expression	(Positive favours One-challenge)<br>TNF-α protein expression: 5.30 (-0.06, 10.66)<br>(Positive favours challenge)<br>IL-6 protein expression: 2.20 (-0.46, 4.86)<br><br><br>(Negative favours One-challenge)<br>TNF-α protein expression: -2.35 (-5.13, 0.43)<br><br>(Negative favours One-challenge)<br>IL-6 protein expression: -7.35 (-14.97, -0.08)
17	Ferlito *et al.* (2001)	THP-1 (human monocyte)<br>(n = 3)	1 µg/ml LPS (not mentioned bacterial origin)<br>1 µg/ml LPS, 10 µg/ml LPS	18h<br><br><br>18h (1st), 18h (2nd)	Group 1: One-challenge<br>Group 2: Control<br>Group 1: Two-challenges<br>Group 2: One-challenge	TNF-α protein expression<br><br>TNF-α protein expression	(Positive favours One-challenge)<br>TNF-α protein expression: 3.48 (-0.24, 7.19)<br><br>(Negative favours One-challenge)<br>TNF-α protein expression: -5.34 (-10.74, -0.06)
18	Fernandez-Ruiz *et al.* (2014)	Human macrophages<br>(n = 3)	10 ng/ml *Salmonella abortus* LPS	5 days (1st), 1h (2nd)<br><br><br><br>24h<br><br><br>24h<br><br><br>5 days (1st), 24h (2nd)	Group 1: Two-challenges<br>Group 2: One-challenge<br>Group 1: One-challenge<br>Group 2: Control<br><br>Group 1: One-challenge<br>Group 2: Control<br><br>Group 1: Two-challenges<br>Group 2: One-challenge<br><br>Group 1: Two-challenges	IRAK3 mRNA expression<br><br><br><br>TNF-α protein expression<br><br><br>IL-6 protein expression<br><br><br>TNF-α protein expression	(Positive favours Two-challenges)<br>IRAK3 mRNA expression: 2.87 (-0.03, 6.06)<br><br><br><br>(Positive favours One-challenge)<br>TNF-α protein expression: 5.43 (-0.05, 10.91)<br><br><br>(Positive favours One-challenge)<br>IL-6 protein expression: 6.03 (-0.01, 12.08)<br><br><br>(Negative favours One-challenge)<br>TNF-α protein expression: -5.31 (-10.67, 0.06)<br><br><br>(Negative favours One-challenge)

				5 days (1st), 24h (2nd)	Group 2: One-challenge	IL-6 protein expression	IL-6 protein expression: -5.99 (-12.00, 0.01)
19	Finamore *et al*. (2014)	Caco-2/TC7 (Human intestinal cell) (n = 3)	Enterotoxigenic *Escherichia coli*	2.5h	Group 1: One-challenge<br>Group 2: Control	IRAK3 protein expression	Not estimable.
20	Fresno *et al*. (2004)	Human monocytes	0, 0.0005, 0.005, 0.05, 0.5, 1 µM GSNO (NO donor)<br><br>0.5 µM GSNO (NO donor)<br><br>0.5 µM GSNO (NO donor)<br><br>2 ng/ml TNF-α	16h<br><br>2, 6, 8, 16, 24h<br><br>8, 20, 28h<br><br>2, 4, 6, 16, 20, 24h	Group 1: One-challenge<br>Group 2: Control	IRAK3 mRNA expression<br><br>IRAK3 mRNA expression<br><br>IRAK3 protein expression<br><br>IRAK3 mRNA expression	Not estimable.<br><br>Not estimable.<br><br>Not estimable.<br><br>Not estimable.
21	Geng *et al*. (2016)	Mouse bone marrow monocytes	0.1 ng/ml LPS (not mentioned bacterial origin)	5 days	Group 1: One-challenge<br>Group 2: Control	IRAK3 mRNA expression	Not estimable.
22	Gunthner *et al*. (2013)	Human PBMC<br><br>Mouse PBMC	500 ng/ml LPS (not mentioned bacterial origin)<br>500 ng/ml LPS	4, 12, 18 and 24h<br><br>4, 12, 18 and 24h	Group 1: One-challenge<br>Group 2: Control<br>Group 1: One-challenge<br>Group 2: Control	IRAK3 mRNA expression<br><br>IRAK3 mRNA expression	Not estimable.<br><br>Not estimable.
23	Hajishengallis *et al*. (2004)	THP-1 (human monocyte) (n = 3)	10 µg/ml *Porphyromonas gingivalis* LPS	16h	Group 1: One-challenge<br>Group 2: Control	TNF-α protein expression<br>IL-6 protein expression	(Positive favours One-challenge)<br>TNF-α protein expression: 6.44 (0.02, 12.87)<br>IL-6 protein expression: 3.53 (-0.23, 7.30).
24	Han *et al*. (2008)	THP-1 (human monocyte) (n = 3)	0.5 µg/ml *Escherichia coli* 055:B5 LPS<br><br>100 µg/ml *Lactobacillus*	18h<br><br>18h (1st), 4h (2nd)	Group 1: One-challenge<br>Group 2: Control<br><br>Group 1: Two-challenges	NF-κB activation<br><br>TNF-α protein expression	(Positive favours One-challenge)<br>NF-κB activation: 12.20 (0.31, 24.09)<br><br>(Negative favours One-challenge)<br>TNF-α protein expression: -30.97 (-60.91, -1.02)

			plantarum LTA, 0.5 µg/ml LPS	4h	Group 2: One-challenge<br><br>Group 1: One-challenge<br>Group 2: Control	TNF-α protein expression	(Positive favours One-challenge)<br>TNF-α protein expression: 10.10 (0.22, 19.98)
			100 µg/ml *Lactobacillus plantarum* LTA, 0.5 µg/ml LPS	18h (1st), 4h (2nd)	Group 1: IRAK3 silencing/knockout group<br>Group 2: IRAK3 present group.	TNF-α protein expression	Not estimable.
		Mouse bone marrow derived macrophage<br>(n = 3)	100 µg/ml LPS	18h	Group 1: One-challenge<br>Group 2: Control	TNF-α protein expression	(Positive favours One-challenge)<br>TNF-α protein expression: 12.43 (3.77, 21.09)
25	Harada *et al.* (2006)	Human intrahepatic biliary epithelial cell<br>(n = 6)	1 mg/ml *Escherichia coli* 0111:B4 Ultrapure LPS or 300 ng/ml Pam3CSK4 (1st); 1 mg/ml LPS (2nd)	24h (1st), 24h (2nd)	Group 1: One-challenge<br>Group 2: Control	NF-κB activation	Not estimable.
				24h (1st), 24h (2nd)	Group 1: Two-challenges<br>Group 2: One-challenge	NF-κB activation	Not estimable.
			1 µg/ml LPS	3h	Group 1: One-challenge<br>Group 2: Control	IRAK3 mRNA expression	(Positive favours One-challenge)<br>IRAK3 mRNA expression: 6.23 (2.98, 9.47)
26	Hassan *et al.* (2009)	RAW264.7 (mouse macrophage)<br>(n = 3)	1 µg/ml TLR7 agonist (R837) (1st), 100 ng/ml Pam3CysSK4 TLR2 ligand (2nd)	18h (1st), 6h (2nd)	Group 1: Two-challenges<br>Group 2: One-challenge		Not estimable.
			100 ng/ml Pam3CysSK4 TLR2 ligand	6h	Group 1: One-challenge<br>Group 2: Control	TNF-α protein expression	(Positive favours One-challenge)<br>TNF-α protein expression: 8.36 (0.13, 16.58)
			1 µg/ml TLR7 agonist (R837) () (1st), 100 ng/ml	18h (1st), 6h (2nd)	Group 1: IRAK3 silencing/knockout group	TNF-α protein expression	Not estimable.

			Pam3CysSK4 TLR2 ligand (2nd)		Group 2: IRAK3 present group		
27	Hayashi *et al*. (2009)	Mouse bone-marrow derived macrophages	100 nM TLR7 agonist (1V136)<br><br>100 nM TLR7 agonist (1V136)	5min, 30min, 2h, 24h<br><br>24h (1st); 5min, 30min, 2h, 24h (2nd)	Group 1: One-challenge<br>Group 2: Control<br>Group 1: Two-challenges<br>Group 2: One-challenge	IRAK3 protein expression<br><br>IRAK3 protein expression	Not estimable.<br><br>Not estimable.
28	Ho *et al*. (2020)	Intestinal porcine enterocytes IPEC-J2 cells	1μg/mL LPS (*E. coli* O55:B5) (Sigma-Aldrich) for (n = 6)	24h	Group 1: One-challenge<br>Group 2: Control	IRAK3 mRNA expression<br><br>IL-6 protein expression	Not estimable.<br><br>(Positive favours One-challenge)<br>IL-6 protein expression: 2.06 (0.55, 3.57)
29	Hoogerwerf *et al*. (2012)	Mouse alveolar macrophages (n = 4)	10 μg/ml *Klebsiella* LPS	16h	Group 1: One-challenge<br>Group 2: Control<br><br>Group 1: IRAK3 silencing/knockout<br>Group 2: IRAK3 present<br><br>Group 1: IRAK3 silencing/knockout<br>Group 2: IRAK3 present	TNF-α protein expression<br><br>IL-6 protein expression<br><br>TNF-α protein expression<br><br>IL-6 protein expression	(Positive favours One-challenge)<br>TNF-α protein expression: 12.43 (3.77, 21.09)<br><br>(Positive favours One-challenge)<br>IL-6 protein expression: 3.84 (0.86, 6.83)<br><br>(Positive favours IRAK3 present)<br>TNF-α protein expression: 5.75 (1.56, 9.94)<br><br>Not estimable.
30	Im *et al*. (2015)	Human periodontal ligament (PDL) cells	0, 7.5, 15 or 30 μg/ml *Staphylococcus aureus*, *Bacillus subtilis* or	1h (1st), 24h (2nd)	Group 1: One-challenge<br>Group 2: Control	NF-κB activity<br><br>NF-κB activity	Not estimable.<br><br>Not estimable.

			Enterococcus faecalis LTA (1st), 0.1 µg/ml *Aggregatibacter actinomycetemcomitans* LPS (2nd).		Group 1: Two-challenges<br>Group 2: One-challenge		
31	Im *et al*. (2020)	Human periodontal ligament (PDL) cells (n = 1)	1 ug/mL *P. gingivalis* LPS	3h	Group 1: One-challenge<br>Group 2: Control	IRAK3 protein expression	Not estimable.
32	Jiang *et al*. (2017)	Mouse bronchoalveolar lavage macrophages (n = 5)	100 ng/ml LPS (not mentioned bacterial origin)	24h	Group 1: One-challenge<br>Group 2: Control<br><br>Group 1: IRAK3 silencing/knockout<br>Group 2: IRAK3 present	TNF-α protein expression<br><br>TNF-α protein expression	(Positive favours One-challenge)<br>TNF-α protein expression: 11.04 (4.70, 17.38)<br><br>(Positive favours IRAK3 silencing/knockout)<br>TNF-α protein expression: 2.82 (0.80, 4.83)
33	Julian *et al*. (2015)	Flt3L-expanded mouse splenocytes (n = 4)	10 ng/ml *Escherichia coli* 0111:B4 LPS<br><br>10 ng/ml LPS	24h<br><br>24h (1st), 24h (2nd).	Group 1: One-challenge<br>Group 2: Control<br><br>Group 1: Two-challenges<br>Group 2: One-challenge	TNF-α protein expression<br><br>TNF-α protein expression	(Positive favours One-challenge)<br>TNF-α protein expression: 38.85 (12.43, 65.61)<br><br>(Positive favours One-challenge)<br>TNF-α protein expression: -49.87 (-83.87, -15.48)
34	Kanakaraj *et al*. (1998)	Mouse skin and embryonic fibroblasts	0.01, 0.1, 1, 10 ng/ml IL-1	8h	Group 1: One-challenge<br>Group 2: Control	IL-6 protein expression	Not estimable.
35	Kim *et al*. (2008)	RAW264.7 (mouse macrophage) (n = 3)	50 ng/ml *Salmonella minnesota* Re 595 LPS	12h<br><br>6h	Group 1: One-challenge<br>Group 2: Control<br><br>Group 1: One-challenge	NF-κB activation<br><br>TNF-α protein expression	(Positive favours One-challenge)<br>NF-kB activation: 3.77 (-0.21, 7.75)<br><br>(Positive favours One-challenge)<br>TNF-α protein expression: 4.36 (-0.14, 8.86)

					Group 2: Control		
36	Kim *et al*. (2012)	THP-1 (human monocyte) (n = 3)	1 – 10 µg/ml *Escherichia coli* 055:B5 LPS	6h<br><br><br><br>24h<br><br><br><br>3h<br><br><br><br>24h (1st); 6h (2nd)	Group 1: One-challenge Group 2: Control<br><br>Group 1: One-challenge Group 2: Control<br><br>Group 1: One-challenge Group 2: Control<br><br>Group 1: Two-challenges Group 2: One-challenge	TNF-α protein expression<br><br><br><br>TNF-α protein expression<br><br><br><br>TNF-α protein expression<br><br><br><br>TNF-α protein expression	(Positive favours One-challenge) TNF-α protein expression: 9.99 (0.21, 19.77)<br><br><br>(Positive favours One-challenge) TNF-α protein expression: 6.77 (0.04, 13.51)<br><br><br>Not estimable<br><br><br><br>(Negative favours One-challenge) TNF-α protein expression: -0.32 (-1.95, 1.31)
37	Kobayashi *et al*. (2002)	Mouse bone marrow-derived macrophages (n = 3)	10 ng/ml *Salmonella abortus* LPS	24h	Group 1: One-challenge Group 2: Control<br><br>Group 1: One-challenge Group 2: Control<br><br>Group 1: IRAK3 silencing/knockout Group 2: IRAK3 present<br><br>Group 1: IRAK3 silencing/knockout Group 2: IRAK3 present	TNF-α protein expression<br><br><br><br>IL-6 protein expression<br><br><br><br>TNF-α protein expression<br><br><br><br><br>IL-6 protein expression	(Positive favours One-challenge) TNF-α protein expression: 20.68 (37.31, 201.81)<br><br>(Positive favours One-challenge) IL-6 protein expression: 18.44 (0.56, 36.32)<br><br><br>(Positive favours IRAK3 silencing/knockout) TNF-α protein expression: 0.41 (-1.24, 2.06)<br><br><br><br>Not estimable.
38	Lagler *et al*. (2009)	Mouse alveolar macrophages (n = 3)	2 x 10^7 CFU/ml or 10^6 CFU/ml *Streptococcus pneumoniae*	6h	Group 1: One-challenge Group 2: Control	IRAK3 mRNA expression	(Positive favours One-challenge) IRAK3 mRNA expression: 20.68 (37.31, 201.81)

39	Lee *et al.* (2013)	Human fibroblast-like synoviocytes	10 ng/ml TNF-α	0, 6, 24, 48, 72, 96h	Group 1: One-challenge<br>Group 2: Control	TNF-α protein expression	Not estimable.
40	Li *et al.* (2009)	Mouse PBMCs<br>(n = 3)	100 ng/ml *Escherichia coli* 026:B6 LPS	1h	Group 1: One-challenge<br>Group 2: Control	IRAK3 mRNA expression	(Negative favours control)<br>IRAK3 mRNA expression: -0.51 (-2.18, 1.17)
				24h	Group 1: One-challenge<br>Group 2: Control	IRAK3 mRNA expression	(Negative favours control)<br>IRAK3 mRNA expression: -0.08 (-1.68, 1.52)
			10 ng/ml, 100 ng/ml *Escherichia coli* 026:B6 LPS	24h (1st), 1h (2nd)	Group 1: Two-challenges<br>Group 2: One-challenge	IRAK3 mRNA expression	(Positive favours Two-challenges)<br>IRAK3 mRNA expression: 1.76 (-0.57, 4.10)
41	Liu *et al.* (2008)	Mouse Kupffer cells<br>(n = 3)	10 ng/ml LPS (not mentioned bacterial origin), 100 ng/ml LPS	24h (1st), 3h (2nd)	Group 1: Two-challenges<br>Group 2: One-challenge	IRAK3 mRNA expression<br>IRAK3 protein expression	(Positive favours Two-challenges)<br>IRAK3 mRNA expression: 7.30 (0.07, 14.52)<br>Not estimable.
			10 ng/ml LPS, 100 ng/ml LPS	24h (1st), 3h (2nd)	Group 1: Two-challenges<br>Group 2: One-challenge	TNF-α protein expression	(Negative favours One-challenge)<br>TNF-α protein expression: -8.04 (-15.96, -0.11)
			10 ng/ml LPS, 100 ng/ml LPS	24h (1st), 3h (2nd)	Group 1: IRAK3 silencing/knockout<br>Group 2: IRAK3 present	TNF-α protein expression<br><br>NF-κB activity	Not estimable.<br><br>Not estimable.
42	Liu *et al.* (2016)	BV2 (murine microglial cell line)<br>(n = 3)	1 μg/ml *Escherichia coli* 055: B5 LPS	6h	Group 1: One-challenge<br>Group 2: Control	IL-6 protein expression	(Positive favours One-challenge)<br>IL-6 protein expression: 37.60 (1.26, 73.95)
				3h	Group 1: One-challenge<br>Group 2: Control	IL-6 protein expression	(Positive favours One-challenge)<br>IL-6 protein expression: 19.48 (0.60, 38.37)
			100 ng/ml LPS, 1 μg/ml LPS	18h (1st), 12h (2nd)	Group 1: Two-challenges	IL-6 protein expression	(Negative favours One-challenge)<br>IL-6 protein expression: -19.81 (-39.01, -0.61)

					Group 2: One-challenge		
43	Lunz *et al*. (2007)	Mouse liver dendritic cells (n = 3)	100 ng/ml LPS (Sigma-Aldrich, not mentioned bacterial origin)	48h	Group 1: One-challenge<br>Group 2: Control	IL-6 protein expression	(Positive favours One-challenge)<br>IL-6 protein expression: 0.79 (-0.99, 2.56)
	Lyroni *et al*. (2017)	RAW264.7 (mouse macrophage) (n = 3)	100 ng/ml *Escherichia coli* O111:B4 LPS	2h<br><br><br>6h<br><br><br>24h<br><br><br>12h<br><br><br>24h	Group 1: One-challenge<br>Group 2: Control<br>Group 1: One-challenge<br>Group 2: Control<br>Group 1: One-challenge<br>Group 2: Control<br>Group 1: One-challenge<br>Group 2: Control<br>Group 1: One-challenge<br>Group 2: Control	IRAK3 mRNA expression<br><br>IRAK3 mRNA expression<br><br>IRAK3 mRNA expression<br><br>IRAK3 protein expression<br><br>IRAK3 protein expression	Not estimable.<br><br><br>(Positive favours One-challenge)<br>IRAK3 mRNA expression: 2.98 (-0.31, 6.26)<br><br>(Positive favours One-challenge)<br>IRAK3 mRNA expression: 14.73 (0.42, 29.05)<br>Not estimable.<br><br><br>Not estimable.
44	Maldifassi *et al*. (2014)	Human macrophages (n = 5)	100 ng/ml LPS (not mentioned bacterial origin)	24h<br><br><br><br>8h (1st), 24h (2nd)<br><br><br><br><br>8h<br><br><br><br>24h	Group 1: One-challenge<br>Group 2: Control<br><br>Group 1: Two-challenges<br>Group 2: One-challenge<br><br>Group 1: One-challenge<br>Group 2: Control<br><br>Group 1: One-challenge<br>Group 2: Control	IRAK3 protein expression<br><br><br><br>IRAK3 protein expression<br><br><br><br><br>TNF-α protein expression<br><br><br>TNF-α protein expression<br>TNF-α protein expression	(Positive favours One-challenge)<br>IRAK3 protein expression: 2.96 (0.88, 5.03)<br><br><br>Not estimable.<br><br><br><br><br>(Positive favours One-challenge)<br>TNF-α protein expression: 24.10 (10.48, 37.73)<br><br>(Positive favours One-challenge)<br>TNF-α protein expression: 4.28 (1.57, 6.99)<br><br>(Negative favours One-challenge)

				8h (1st), 24h (2nd)	Group 1: Two-challenges<br>Group 2: One-challenge		TNF-α protein expression: -19.79 (-31.01, -8.58)
45	Mandrekar *et al*. (2009)	RAW264.7 (mouse macrophage)<br>(n = 3)	100 ng/ml *Echerichia coli* 0111:B4 LPS	6h	Group 1: One-challenge<br>Group 2: Control<br><br>Group 1: IRAK3 silencing/knockout group<br>Group 2: IRAK3 present group	TNF-α protein expression	(Positive favours One-challenge)<br>TNF-α protein expression: 19.00 (0.58, 37.41)<br><br>Not estimable.
		Human monocytes	100 ng/ml LPS	6h	Group 1: One-challenge<br>Group 2: Control	IRAK3 mRNA expression	(Positive favours One-challenge)<br>IRAK3 mRNA expression: 0.86 (-0.64, 2.37)
46	Mansilla *et al.* (2020)	Bovine intestinal epithelial cells line (BIE cells)	*Lactobacillus acidophilus* for 48h, heat-stable *E. coli* pathogen-associated molecular patterns	48h (1st), 12h (2nd)	Group 1: Two-challenges<br>Group 2: One-challenge	IRAK3 mRNA expression	(Positive favours Two-challenges)<br>IRAK3 mRNA expression: 2.29 (-0.44, 5.03)
47	Miyata *et al*. (2015)	Human primary macrophages	*Haemophilus influenzae*	5h	Group 1: One-challenge.<br>Group 2: Control	IRAK3 mRNA expression	(Positive favours One-challenge)<br>IRAK3 mRNA expression: 6.40 (0.02, 12.78)
48	Nakayama *et al*. (2004)	RAW264.7 (mouse macrophage)<br>(n = 3)	30 µg/ml Peptidoglycan	16h<br><br><br>16h<br><br><br><br>16h (1st)<br>24h (2nd).	Group 1: One-challenge<br>Group 2: Control<br><br>Group 1: IRAK3 silencing/knockout group<br>Group 2: IRAK3 present group<br>Group 1: IRAK3 silencing/knockout group	TNF-α protein expression	(Positive favours One-challenge)<br>TNF-α protein expression: 9.55 (0.19, 18.90)<br><br>(Negative favours IRAK3 present)<br>TNF-α protein expression: -1.75 (-4.08, 0.58)<br><br>Not estimable.

					Group 2: IRAK3 present group<br>Group 1: Two-challenges group<br>Group 2: One-challenge		(Negative favours One-challenges)<br>TNF-α protein expression: -7.40 (-14.72, -0.08)
49	Nguyen *et al*. (2012)	Human alveolar macrophages	10 μg/ml Surfactant protein A and 100 μg/ml Survantan	1h – 6h – 12h – 24h	Group 1: One-challenge<br>Group 2: Control	IRAK3 mRNA expression	Not estimable.
50	Odoms *et al*. (2004)	BEAS-2B (human lung epithelial cell)	Hyperoxia alone, IL-1β, and hyperoxia and IL-1β	0.5h – 1h – 2h – 3h	Group 1: One-challenge<br>Group 2: Control	DNA binding of NF-kB	Not estimable.
51	Paik *et al*. (2019)	Mouse BMDMs (n = 4)	100 ng/ml *Escherichia coli* O26:B6 LPS<br><br>100 ng/ml LPS	18h<br><br>6h	Group 1: One-challenge<br>Group 2: Control<br><br>Group 1: One-challenge<br>Group 2: Control	TNF-α protein expression<br>NF-κB activity<br><br>IRAK3 mRNA expression	(Positive favours One-challenge)<br>TNF-α protein expression: 7.98 (2.32, 13.64)<br>Not estimable.<br><br>(Positive favours One-challenge)<br>IRAK3 mRNA expression: 4.95 (1.28, 8.63)
52	Parmar *et al*. (2019)	J774 (mouse macrophages) (n = 3)	100 ng/ml LPS (not mentioned bacterial origin)<br><br>Infected with *Leishmania donovani* promastigotes	24h<br><br>24h	Group 1: One-challenge<br>Group 2: Control<br><br>Group 1: IRAK3 silencing/knockout<br>Group 2: IRAK3 present	TNF-α protein expression<br><br>IL-6 protein expression<br><br>TNF-α protein expression<br><br>NF-κB activity	(Positive favours One-challenge)<br>TNF-α protein expression: 16.40 (0.48, 32.32)<br><br>(Positive favours One-challenge)<br>IL-6 protein expression: 13.35 (0.36, 26.34)<br><br>(Positive favours IRAK3 silencing/knockout)<br>TNF-α protein expression: 10.48 (0.23, 20.73)<br><br>Not estimable.
53	Peck *et al*. (2004)	THP-1 (human monocyte) (n = 3)	100 ng/ml *Salmonella Enteritidis* LPS	24h	Group 1: One-challenge<br>Group 2: Control	TNF-α protein expression	(Positive favours One-challenge)<br>TNF-α protein expression: 2.69 (-0.36, 5.73)

				24h (1st), 24h (2nd)	Group 1: Two-challenges group<br>Group 2: One-challenge	TNF-α protein expression	(Negative favours One-challenge)<br>TNF-α protein expression: -2.73 (-5.82, 0.35)
54	Petricevic *et al*. (2009)	Human monocytes<br>(n = 3)	100 ng/ml *Escherichia coli* O55:B5 LPS	24h<br><br><br><br><br>24h (1st), 24h (2nd)	Group 1: One-challenge<br>Group 2: Control<br><br><br>Group 1: Two-challenges group<br>Group 2: One-challenge	IRAK3 protein expression<br>TNF-α protein expression<br><br>TNF-α protein expression	(Positive favours One-challenge)<br>IRAK3 protein expression: 1.79 (0.36, 3.22)<br>(Positive favours challenge)<br>TNF-α protein expression: 14.51 (0.41, 28.62)<br><br>(Positive favours One-challenge)<br>TNF-α protein expression: -5.35 (-10.75, 0.06)
55	Rajaiah *et al*. (2013)	Mouse macrophage<br>(n = 3)	100 ng/ml *Escherichia coli* K235 LPS	24 h (1st), 3 h (2nd)	Group 1: Two-challenges<br>Group 2: One-challenge	IRAK3 mRNA expression	(Positive favours Two-challenges)<br>IRAK3 mRNA expression: 4.81 (-0.10, 9.73)
56	Saito *et al*. (2013)	Mouse BMDCs<br>(n = 3)	10 ng/ml *Escherichia coli* LPS	24h (1st), 24h (2nd).	Group 1: Two-challenges<br>Group 2: One-challenge	TNF-α protein expression<br><br>IL-6 protein expression	(Negative favours One-challenge)<br>TNF-α protein expression: -24.79 (-48.79, -0.80)<br>Not estimable.
57	3 *et al*. (2018)	Human PBMCs (peripheral blood mononuclear cells)<br>(n = 15)	10 µg/ml *Escherichia coli* 0111: B4 LPS<br><br>10 µg/ml LPS	18h<br><br><br>18h	Group 1: One-challenge<br>Group 2: Control<br>Group 1: One-challenge<br>Group 2: Control	IRAK3 mRNA expression<br><br>TNF-α protein expression	(Positive favours One-challenge)<br>IRAK3 mRNA expression: 1.32 (0.52, 2.12)<br><br>(Positive favours One-challenge)<br>TNF-α protein expression: 0.44 (-0.81, 1.69)
58	Sanaei *et al*. (2019)	Human PBMCs (peripheral blood mononuclear cells) (control group n = 15, LPS challenge group n = 14).	1 µg /mL LPS (not mention bacteria origin)	18h – 24h	Group 1: One-challenge<br>Group 2: Control	IRAK3 mRNA expression	(Positive favours One-challenge)<br>IRAK3 mRNA expression: 1.40 (0.58, 2.23)

		Human PBMCs (peripheral blood mononuclear cells) (n = 14).	1 µg /mL LPS (not mention bacteria origin)	18h – 24h	Group 1: One-challenge<br>Group 2: Control	TNF-α protein expression	(Positive favours One-challenge)<br>TNF-α protein expression: 0.61 (-0.15, 1.37)
59	Scotton *et al*. (2005)	Human monocytes	20 ng/ml IL-13	2h - 8h	Group 1: One-challenge<br>Group 2: Control	IRAK3 mRNA expression	Not estimable.
60	Shen *et al*. (2008)	Human PBMC (n = 3)<br><br>Human monocyte derived dendritic cells	0.1 µg/ml LPS (Sigma-Aldrich, not mentioned bacterial origin)<br><br>0.1 µg/ml LPS	24h<br><br>24h	Group 1: One-challenge<br>Group 2: Control<br><br>Group 1: One-challenge<br>Group 2: Control	IRAK3 protein expression<br><br>TNF-α protein expression<br><br>IL-6 protein expression<br><br>NF-κB activation	(Positive favours One-challenge)<br>IRAK3 protein expression: 5.40 (-0.05, 10.85)<br>(Positive favours One-challenge)<br>TNF-α protein expression: 34.59 (1.15, 68.03)<br><br>(Positive favours One-challenge)<br>IL-6 protein expression: 10.54 (0.24, 20.83)<br><br>Not estimable.
61	Shiu *et al*. (2013)	Mouse BMDCs (n = 3)	*Helicobacter pylori* antigen	8h<br><br>24h<br><br>8h<br><br>24h	Group 1: One-challenge<br>Group 2: Control<br>Group 1: One-challenge<br>Group 2: Control<br><br>Group 1: IRAK3 silencing/knockout<br>Group 2: IRAK3 present<br>Group 1: IRAK3 silencing/knockout<br>Group 2: IRAK3 present	TNF-α protein expression<br><br>TNF-α protein expression<br><br>TNF-α protein expression<br><br>TNF-α protein expression	(Positive favours One-challenge)<br>TNF-α protein expression: 4.96 (-0.11, 9.49)<br><br>(Positive favours One-challenge)<br>TNF-α protein expression: 7.83 (0.10, 15.55)<br><br>Not estimable.<br><br>(Positive favours IRAK3 silencing/knockout)<br>TNF-α protein expression: 1.87 (-0.54, 4.28)

62	Soares-Schanoski, A. *et al*. (2007)	Osteoclasts derived from THP-1 (human monocyte) and RAW264.7 (mouse macrophage)	40 ng/ml Vitamin D3<br><br>0 – 0.4 – 4 – 40 ng/mL Osteoporosis-inducing glucocorticoid, 6-methylprednisolone (6-MP)	1 day – 6 days<br><br>6 days	Group 1: One-challenge<br>Group 2: Control<br>Group 1: One-challenge<br>Group 2: Control	IRAK3 mRNA expression<br><br>IRAK3 mRNA expression	Not estimable.<br><br>Not estimable.
63	Srivastav *et al*. (2015)	RAW264.7 (mouse macrophage)<br>(n = 3)	Infected with *Leishmania donovani* promastigotes	12h<br><br>12h<br><br>3h<br><br>6h<br><br>24h<br><br>3h<br><br>12h	Group 1: One-challenge<br>Group 2: Control<br><br>Group 1: IRAK3 silencing/knockout group<br>Group 2: IRAK3 present group<br>Group 1: One-challenge<br>Group 2: Control<br><br>Group 1: One-challenge<br>Group 2: Control<br><br>Group 1: One-challenge<br>Group 2: Control<br><br>Group 1: One-challenge<br>Group 2: Control<br><br>Group 1: One-challenge<br>Group 2: Control	NF-κB activation<br><br>NF-κB activation<br><br>IRAK3 mRNA expression<br><br>IRAK3 mRNA expression<br><br>IRAK3 mRNA expression<br><br>IRAK3 protein expression<br><br>IRAK3 protein expression<br>TNF-α protein expression	(Positive favours One-challenge)<br>NF-κB activation: 3.77 (-0.21, 7.75)<br><br>Not estimable<br><br>Not estimable.<br><br>(Positive favours One-challenge)<br>IRAK3 mRNA expression: 9.30 (0.18, 18.41)<br><br>(Positive favours One-challenge)<br>IRAK3 mRNA expression: 11.76 (0.29, 23.22)<br><br>Not estimable.<br><br>Not estimable.<br><br>(Negative favours control)<br>TNF-α protein expression: -0.55 (-2.24, 1.14)

				24h	Group 1: One-challenge<br>Group 2: Control	IRAK3 protein expression	Not estimable.
						TNF-α protein expression	(Positive favours One-challenge)<br>TNF-α protein expression: 1.03 (-0.86, 2.91)
				12h	Group 1: IRAK3 silencing/knockout group<br>Group 2: IRAK3 present group	TNF-α protein expression	Not estimable.
				24h	Group 1: IRAK3 silencing/knockout group<br>Group 2: IRAK3 present group	TNF-α protein expression	(Positive favours IRAK3 silencing/knockout)<br>TNF-α protein expression: 8.06 (0.11, 16.00)
64	Standiford *et al*. (2011)	Human PBMCs	10 ng/ml TGF-β	0, 1, 4, 8, 24h	Group 1: One-challenge<br>Group 2: Control	IRAK3 mRNA expression	Not estimable.
65	Stark *et al*. (2016)	Primary human umbilical vein endothelial cells (n = 4)	100 ng/ml *Escherichia coli* 0111:B4 Ultrapure LPS	16h	Group 1: One-challenge<br>Group 2: Control	IRAK3 protein expression	(Positive favours One-challenge)<br>IRAK3 protein expression: 0.19 (-1.20, 1.58)
				16h (1st), 1h (2nd).	Group 1: Two-challenges<br>Group 2: One-challenge	IRAK3 protein expression	Not estimable.
		Primary human umbilical vein endothelial cells (n = 5)		16h	Group 1: One-challenge<br>Group 2: Control	IL-6 protein expression	(Positive favours One-challenge)<br>IL-6 protein expression: 3.96 (1.41, 6.51)
				16h (1st), 6h (2nd).	Group 1: Two-challenges<br>Group 2: One-challenge	IL-6 protein expression	(Negative favours One-challenge)<br>IL-6 protein expression: -10.41 (-16.40, -4.42)

66	Stiehm *et al.* (2013)	Mouse BMDCs (n = 3)	1 ng/ml LPS (not mentioned bacterial origin)	24h	Group 1: One-challenge<br>Group 2: Control	TNF-α protein expression<br><br>IL-6 protein expression	(Positive favours One-challenge)<br>TNF-α protein expression: 9.60 (0.19, 19.00)<br><br>(Positive favours One-challenge)<br>IL-6 protein expression: 5.63 (-0.04, 11.30)
67	Su *et al.* (2007)	THP-1 (human monocyte)	100 ng/ml Pam3CSK4	15min – 1h – 2h – 4h – 8h	Group 1: One-challenge<br>Group 2: Control	IRAK3 protein expression	Not estimable.
68	Su *et al.* (2009)	Mouse BMDMs (n = 3)	100 ng/ml *Escherichia coli* 0114 LPS	12h	Group 1: One-challenge<br>Group 2: Control<br><br>Group 1: IRAK3 silencing/knockout<br>Group 2: IRAK3 present	IL-6 protein expression<br><br>IL-6 protein expression	Not estimable.<br><br>Not estimable.
69	Sun *et al.* (2014)	THP-1 (human monocyte) (n = 5)	1 µg/ml *Porphyromonas gingivalis* LPS and *Escherichia coli* O127:B8 LPS<br>1 µg/ml LPS, 1 µg/ml LPS	24h<br><br>24h (1st), 24h (2nd).	Group 1: One-challenge<br>Group 2: Control<br>Group 1: Two-challenges<br>Group 2: One-challenge	TNF-α protein expression<br><br>TNF-α protein expression	(Positive favours One-challenge)<br>TNF-α protein expression: 21.73 (9.44, 34.03)<br><br>(Negative favours One-challenge)<br>TNF-α protein expression: -16.89 (-33.27, -0.50)
70	Sung *et al.* (2013)	RAW264.7 (mouse macrophage) (n = 3)	50 ng/ml *Escherichia coli* O111:B4 LPS	24h	Group 1: One-challenge<br>Group 2: Control<br><br>Group 1: IRAK3 silencing/knockout group<br>Group 2: IRAK3 present group	TNF-α protein expression<br><br>IL-6 protein expression<br><br>TNF-α protein expression<br>IL-6 protein expression	(Positive favours One-challenge)<br>TNF-α protein expression: 18.19 (0.55, 35.83)<br><br>(Positive favours One-challenge)<br>IL-6 protein expression: 28.98 (0.95, 57.02)<br><br>(Positive favours IRAK3 silencing/knockout)<br>TNF-α protein expression: 0.76 (-1.00, 2.52)<br>Not estimable.
71	Swantek *et al.* (2000)	Mouse macrophages	0.05, 0.1, 0.5, 1, 5, 10 ng/ml *Escherichia coli* K-12 LPS	24h	Group 1: One-challenge<br>Group 2: Control	TNF-α protein expression	Not estimable.

72	Takebayashi *et al*. (2009)	RAW264.7 (mouse macrophage) (n = 3)	10 ng/ml *Escherichia coli* O111:B4 LPS	4h<br><br>4h (1st), 4h (2nd)	Group 1: One-challenge<br>Group 2: Control<br><br>Group 1: Two-challenges<br>Group 2: One-challenge	IRAK3 mRNA expression<br><br>IRAK3 mRNA expression	(Positive favours One-challenge)<br>IRAK3 mRNA expression: 6.06 (-0.01, 12.12)<br><br>(Positive favours Two-challenges)<br>IRAK3 mRNA expression: 3.53 (-0.24, 7.20)
73	Tazi *et al*. (2006)	Human monocyte (n = 11)	100 ng/ml *Escherichia coli* O111:B4 LPS	4h<br><br>24h	Group 1: One-challenge<br>Group 2: Control<br><br>Group 1: One-challenge<br>Group 2: Control	TNF-α protein expression<br><br>TNF-α protein expression	(Positive favours One-challenge)<br>TNF-α protein expression: 4.22 (2.61, 5.83)<br><br>(Positive favours One-challenge)<br>TNF-α protein expression: 3.77 (2.29, 5.26)
74	Tiwari *et al*. (2011)	THP1 (human monocyte)	Paramethoxyamphetamine	0 – 15 – 30 min – 1 – 6 – 12 – 24 – 48h	Group 1: One-challenge<br>Group 2: Control	NF-κB activity<br><br>IRAK3 protein expression	Not estimable.<br><br>Not estimable.
75	Turrel-Davin *et al*. (2011)	Human peripheral blood mononuclear cells (PBMCs) (n = 10)	100 ng/ml *Escherichia coli* O55:B5, O127:B8 and O111:B4 LPS	15h<br><br>15h (1st), 6h (2nd)<br><br>15h<br><br>15h (1st), 6h (2nd)	Group 1: One-challenge<br>Group 2: Control<br><br>Group 1: Two-challenges<br>Group 2: One-challenge<br><br>Group 1: One-challenge<br>Group 2: Control<br><br>Group 1: Two-challenges<br>Group 2: One-challenge	IRAK3 mRNA expression<br><br>IRAK3 mRNA expression<br><br>TNF-α protein expression<br><br>TNF-α protein expression	(Positive favours One-challenge)<br>IRAK3 mRNA expression: 1.79 (0.71, 2.86)<br><br>(Positive favours Two-challenges)<br>IRAK3 mRNA expression: 1.40 (0.40, 2.40)<br><br>(Positive favours One-challenge)<br>TNF-α protein expression: 6.28 (3.94, 8.63)<br><br>(Negative favours One-challenges)<br>TNF-α protein expression: -21.71(-29.27, -14.15)

76	Twayana *et al*. (2019)	Murine microglial BV2 cell line (n = 2)	0.5 µg/ml LPS from Escherichia coli K235 (Sigma Aldrich)	24h	Group 1: One-challenge<br>Group 2: Control	IL-6 protein expression	(Positive favours One-challenge)<br>IL-6 protein expression: 13.36 (-62.24, 88.96)
77	van 't Veer *et al*. (2007)	Human whole blood (n = 3)	0.01 ng/ml *Escherichia coli* Ultrapure LPS	3h<br><br>6h	Group 1: One-challenge<br>Group 2: Control<br><br>Group 1: One-challenge<br>Group 2: Control	IRAK3 mRNA expression	(Positive favours One-challenge)<br>IRAK3 mRNA expression: 0.38 (-1.26, 2.02)<br><br>(Positive favours One-challenge)<br>IRAK3 mRNA expression: 15.95 (0.47, 31.44)
78	Wesche *et al*. (1999)	293 cells (human embryonic kidney epithelial cell) (n = 2)	100 ug/ml LPS (not mentioned bacterial origin) (n = 2)<br>20 ng/ml IL-1β (n = 2)	6h	Group 1: One-challenge<br>Group 2: Control<br><br>Group 1: IRAK3 silencing/knockout group<br>Group 2: IRAK3 present group	NF-κB activation	(Positive favours One-challenge)<br>NF-κB activation: 1.84 (-0.55, 4.24)<br><br>Not estimable
79	Wiersinga *et al*. (2009)	Human whole blood (n = 32)	10 ng/ml *Escherichia coli* 055:B5 LPS	4h	Group 1: One-challenge<br>Group 2: Control	TNF-α protein expression<br><br>IL-6 protein expression	Not estimable.<br><br>Not estimable.
80	Wu *et al*. (2012)	Human lung mucoepidermoid NCIH292 (n = 3)	10 ng/ml Pam3CSK4	6h	Group 1: One-challenge<br>Group 2: Control<br><br>Group 1: IRAK3 silencing/knockout group<br>Group 2: IRAK3 present group	NF-κB p65 activation<br><br>NF-κB p65 activation	Not estimable<br><br>Not estimable

81	Xiong *et al.* (2011)	THP1 cells (human monocyte) (n = 3)	10 ng/ml *Escherichia coli* K23 LPS (1st), 100 ng/ml LPS (2nd)	20h (1st), 24h (2nd)	Group 1: One-challenge<br>Group 2: Control<br><br>Group 1: Two-challenges<br>Group 2: One-challenge	TNF-α protein expression<br><br><br>TNF-α protein expression	(Positive favours One-challenge)<br>TNF-α protein expression: 30.76 (1.01, 60.51)<br><br>(Negative favours One-challenges)<br>TNF-α protein expression: -4.80 (-9.70, 0.10)
82	Xiong *et al.* (2013)	Mouse peripheral macrophage (n = 3)	10 ng/ml *Escherichia coli* K235 LPS	20h<br><br><br><br>20h (1st), 24h (2nd)	Group 1: One-challenge<br>Group 2: Control<br><br>Group 1: Two-challenges<br>Group 2: One-challenge	TNF-α protein expression<br><br><br>TNF-α protein expression	(Negative favours control)<br>TNF-α protein expression: -0.45 (-2.10, 1.21)<br><br>(Negative favours One-challenge)<br>TNF-α protein expression: -17.42 (-34.32, -0.52)
83	Yee and Hamerman (2013)	Mouse BMDMs (n = 3)	1 ng/ml *Salmonella Minnesota* R595 Ultrapure LPS<br><br>1 ng/ml *Escherichia coli* 0111:B4 LPS	8h<br><br><br><br>0, 4, 8, 12h	Group 1: One-challenge<br>Group 2: Control<br><br>Group 1: One-challenge<br>Group 2: Control	IRAK3 mRNA expression<br><br><br>TNF-α protein expression	(Positive favours One-challenge)<br>IRAK3 mRNA expression: 4.52 (-0.13, 9.16)<br><br>Not estimable.
85	Zhang *et al.* (2012)	RAW264.7 (mouse macrophage)	0.0001, 0.001, 0.01, 0.1, and 1 mg/ml of Ti particles or bone cement	0, 0.5, 1, 3, 6, 9, 12, and 24h.	Group 1: One-challenge<br>Group 2: Control	IRAK3 mRNA expression	Not estimable.
86	Zhang *et al.* (2013)	RAW264.7 (mouse macrophage)	100 ng/ml *Escherichia coli* 0111:B4 LPS, Ti particles	0.5, 1, 3, 6, 12, and 24h.<br><br><br>12h (1st), 24h (2nd)	Group 1: One-challenge<br>Group 2: Control<br><br>Group 1: IRAK3 silencing/knockout<br>Group 2: IRAK3 present	TNF-α protein expression<br><br><br>TNF-α protein expression	Not estimable.<br><br><br><br>Not estimable.
87	Zhou *et al.* (2013)	Mouse BMDMs	1 μg/ml R848 (TLR7 ligand)	0, 0.5, 1, 2, 4h	Group 1: One-challenge	TNF-α, IL-6 protein expression	Not estimable.

					Group 2: Control<br><br>Group 1: IRAK3 silencing/knockout<br>Group 2: IRAK3 present		 <br>Not estimable.
88	Zhou *et al*. (2016)	Mouse BMDMs (n = 3)	1 µg/ml LPS (not mentioned bacterial origin)	8h<br><br><br><br><br><br>24h	Group 1: One-challenge<br>Group 2: Control	TNF-α protein expression<br><br>IL-6 protein expression<br><br>TNF-α protein expression<br>IL-6 protein expression	(Positive favours One-challenge)<br>TNF-α protein expression: 34.58 (15.07, 54.08)<br>Not estimable.<br><br><br>(Positive favours One-challenge)<br>TNF-α protein expression: 6.64 (2.70, 10.58)<br>(Positive favours One-challenge)<br>IL-6 protein expression: 15.91 (6.87, 24.95)
89	Zhou *et al*. (2018)	Mouse BMDCs (n = 3)	1 µg/ml LPS (not mentioned bacterial origin) (1st); 4 µg/ml LPS (2nd)	48h (1st), 48h (2nd)	Group 1: Two-challenges<br>Group 2: One-challenge	IL-6 protein expression	Not estimable.

S3 Table. List of excluded studies.

**Reasons for exclusion**	**Articles**
Not relevant data	1. Al-Qahtani AA, Lyroni K, Aznaourova M, Tseliou M, Al-Anazi MR, Al-Ahdal MN, *et al*. Middle east respiratory syndrome corona virus spike glycoprotein suppresses macrophage responses via DPP4-mediated induction of IRAK-M and PPARγ. *Oncotarget*. (2017) 8(6):9053-66. doi: 10.18632/oncotarget.14754.<br>2. Balaci L, Spada MC, Olla N, Sole G, Loddo L, Anedda F, *et al*. IRAK-M is involved in the pathogenesis of early-onset persistent asthma. *Am J Hum Genet*. (2007) 80(6):1103-14. doi: 10.1016/518259.<br>3. Dong GH, Gong JP, Li JZ, Luo YH, Li ZD, Li PZ, *et al*. Association between gene polymorphisms of IRAK-M and the susceptibility of sepsis. *Inflammation*. (2013) 36(5):1087-93. doi: 10.1007/s10753-013-9641-z.<br>4. Gribar SC, Sodhi CP, Richardson WM, Anand RJ, Gittes GK, Branca MF, *et al*. Reciprocal expression and signaling of TLR4 and TLR9 in the pathogenesis and treatment of necrotizing enterocolitis. *J Immunol*. (2009) 182(1):636-46. doi: 10.4049/jimmunol.182.1.636.<br>5. Hulsmans M, Geeraert B, De Keyzer D, Mertens A, Lannoo M, Vanaudenaerde B, *et al*. Interleukin-1 receptor-associated kinase-3 is a key inhibitor of inflammation in obesity and metabolic syndrome. *PLoS One*. (2012) 7(1):e30414. doi: 10.1371/journal.pone.0030414.

6. Hulsmans M, Van Dooren E, Mathieu C, Holvoet P. Decrease of miR-146b-5p in monocytes during obesity is associated with loss of the anti-inflammatory but not insulin signaling action of adiponectin. *PLoS One*. (2012) 7(2):e32794. doi: 10.1371/journal.pone.0032794.

7. Jin P, Bo L, Liu Y, Lu W, Lin S, Bian J, *et al*. Activator protein 1 promotes the transcriptional activation of IRAK-M. *Biomed Pharmacother*. (2016) 83:1212-9. doi: 10.1016/j.biopha.2016.08.024.

8. Kim YI, Park JE, Kwon KH, Hong CY, Yi AK. Interleukin-1 receptor-associated kinase 2- and protein kinase D1-dependent regulation of IRAK-monocyte expression by CpG DNA. *PLoS One*. (2012) 7(8). doi: 10.1371/journal.pone.0043970.

9. Kobayashi H, Nolan A, Naveed B, Hoshino Y, Segal LN, Fujita Y, *et al*. Neutrophils activate alveolar macrophages by producing caspase-6-mediated cleavage of IL-1 receptor-associated kinase-M. *J Immunol*. (2011) 186(1):403-10. doi: 10.4049/jimmunol.1001906.

10. Learn CA, Boger MS, Li L, McCall CE. The phosphatidylinositol 3-kinase pathway selectively controls sIL-1RA not interleukin-1β production in the septic leukocytes. *J Biol Chem*. (2001) 276(23):20234-9. doi: 10.1074/jbc.M100316200.

11. Lee SA, Fitzgerald SM, Huang SK, Li C, Chi DS, Milhorn DM, *et al*. Molecular regulation of interleukin-13 and monocyte chemoattractant protein-1 expression in human mast cells by interleukin-1β. *Am J Respir Cell Mol Biol*. (2004) 31(3):283-91. doi: 10.1165/rcmb.2004-0089OC.

12. Li L, Cousart S, Hu J, McCall CE. Characterization of interleukin-1 receptor-associated kinase in normal and endotoxin-tolerant cells. *J Biol Chem*. (2000) 275(30):23340-5. doi: 10.1074/jbc.M001950200.

13. Lu X, Xue L, Sun W, Ye J, Zhu Z, Mei H. Identification of key pathogenic genes of sepsis based on the gene expression Omnibus database. *Mol Med Rep*. (2018) 17(2):3042-54. doi: 10.3892/mmr.2017.8258.

14. Maitra U, Li L. Molecular mechanisms responsible for the reduced expression of cholesterol transporters from macrophages by low-dose endotoxin. *Arterioscler Thromb Vasc Biol*. (2013) 33(1):24-33. doi: 10.1161/atvbaha.112.300049.

15. Nanthakumar N, Meng D, Goldstein AM, Zhu W, Lu L, Uauy R, *et al*. The mechanism of excessive intestinal inflammation in necrotizing enterocolitis: An immature innate immune response. *PLos One*. (2011) 6(3): e17776. doi: 10.1371/journal.pone.0017776.

16. Patenaude J, D'Elia M, Côté-Maurais G, Bernier J. LPS response and endotoxin tolerance in Flt-3L-induced bone marrow-derived dendritic cells. *Cell Immunol*. (2011) 271(1):184-91. doi: 10.1016/j.cellimm.2011.06.020.

17. Pathak SK, Basu S, Bhattacharyya A, Pathak S, Kundu M, Basu J. *Mycobacterium tuberculosis* lipoarabinomannan-mediated IRAK-M induction negatively regulates Toll-like receptor-dependent interleukin-12 p40 production in macrophages. *J Biol Chem*. (2005) 280(52):42794-800. doi: 10.1074/jbc.M506471200.

18. Peng Q, O'Loughlin JL, Humphrey MB. DOK3 negatively regulates LPS responses and endotoxin tolerance. *PLoS One*. (2012) 7(6):e39967. doi: 10.1371/journal.pone.0039967.

19. Pino-Yanes M, Ma SF, Sun X, Tejera P, Corrales A, Blanco J, *et al*. Interleukin-1 receptor-associated kinase 3 gene associates with susceptibility to acute lung injury. *Am J Respir Cell Mol Biol*. (2011) 45(4):740-5. doi: 10.1165/rcmb.2010-0292OC.

20. Shalova IN, Kajiji T, Lim JY, Gomez-Pina V, Fernandez-Ruiz I, Arnalich F, *et al*. CD16 regulates TRIF-dependent TLR4 response in human monocytes and their subsets. *J Immunol*. (2012) 188(8):3584-93. doi: 10.4049/jimmunol.1100244.

21. Sumpter TL, Packiam V, Turnquist HR, Castellaneta A, Yoshida O, Thomson AW. DAP12 promotes IRAK-M expression and IL-10 production by liver myeloid dendritic cells and restrains their T cell allostimulatory ability. *J Immunol*. (2011) 186(4):1970-80. doi: 10.4049/jimmunol.1000527.

	22. Taylor AW. The immunomodulating neuropeptide alpha-melanocyte-stimulating hormone (α-MSH) suppresses LPS-stimulated TLR4 with IRAK-M in macrophages. *J Neuroimmunol*. (2005) 162(1-2):43-50. doi: 10.1016/j.neuroim.2005.01.008.<br>23. Woehrle T, Du W, Goetz A, Hsu H-Y, Joos TO, Weiss M, *et al*. Pathogen specific cytokine release reveals an effect of TLR2 Arg753Gln during *Candida* sepsis in humans. *Cytokine*. (2008) 41(3):322-9. doi: 10.1016/j.cyto.2007.12.006.<br>24. Yang Q, Calvano SE, Lowry SF, Androulakis IP. A dual negative regulation model of Toll-like receptor 4 signaling for endotoxin preconditioning in human endotoxemia. *Math Biosci*. (2011) 232(2):151-63. doi: 10.1016/j.mbs.2011.05.005.<br>25. Zhang FX, Kirschning CJ, Mancinelli R, Xu XP, Jin Y, Faure E, *et al*. Bacterial lipopolysaccharide activates nuclear factor-κB through interleukin-1 signaling mediators in cultured human dermal endothelial cells and mononuclear phagocytes. *J Biol Chem*. (1999) 274(12):7611-4. doi: 10.1074/jbc.274.12.7611.
In vivo studies	26. Ballinger MN, Newstead MW, Zeng X, Bhan U, Horowitz JC, Moore BB, *et al*. TLR signaling prevents hyperoxia-induced lung injury by protecting the alveolar epithelium from oxidant-mediated death. *J Immunol*. (2012) 189(1):356-64. doi: 10.4049/jimmunol.1103124.<br>27. Berglund M, Melgar S, Kobayashi KS, Flavell RA, Hornquist EH, Hultgren OH. IL-1 receptor-associated kinase M downregulates DSS-induced colitis. *Inflamm Bowel Dis*. (2010) 16(10):1778-86. doi: 10.1002/ibd.21287.<br>28. Deng JC, Cheng G, Newstead MW, Zeng X, Kobayashi K, Flavell RA, *et al*. Sepsis-induced suppression of lung innate immunity is mediated by IRAK-M. *J Clin Invest*. (2006) 116(9):2532-42. doi: 10.1172/jci28054.<br>29. Gong H, Liu T, Chen W, Zhou W, Gao J. Effect of IRAK-M on Airway Inflammation Induced by Cigarette Smoking. *Mediat Inflamm*. (2017) 2017:6506953-. doi: 10.1155/2017/6506953.<br>30. Hubbard LL, Ballinger MN, Thomas PE, Wilke CA, Standiford TJ, Kobayashi KS, *et al*. A role for IL-1 receptor-associated kinase-M in prostaglandin E2-induced immunosuppression post-bone marrow transplantation. *J Immunol*. (2010) 184(11):6299-308. doi: 10.4049/jimmunol.0902828.<br>31. Kallapur SG, Jobe AH, Ball MK, Nitsos I, Moss TJM, Hillman NH, *et al*. Pulmonary and systemic endotoxin tolerance in preterm fetal sheep exposed to chorioamnionitis. *J Immunol*. (2007) 179(12):8491-9. doi: 10.4049/jimmunol.179.12.8491.<br>32. Klimesova K, Kverka M, Zakostelska Z, Hudcovic T, Hrncir T, Stepankova R, et al. Altered gut microbiota promotes colitis-associated cancer in IL-1 receptor-associated kinase M-deficient mice. *Inflamm Bowel Dis*. (2013) 19(6):1266-77. doi: 10.1097/MIB.0b013e318281330a.<br>33. Lech M, Grobmayr R, Ryu M, Lorenz G, Hartter I, Mulay SR, *et al*. Macrophage phenotype controls long-term AKI outcomes-kidney regeneration versus atrophy. *J Am Soc Nephrol*. (2014) 25(2):292-304. doi: 10.1681/ASN.2013020152.<br>34. Li S, Luo C, Yin C, Peng C, Han R, He Q, *et al*. Endogenous HMGB1 is required in endotoxin tolerance. *J Surg Res*. (2013) 185(1):319-28. doi: 10.1016/j.jss.2013.05.062.<br>35. Liu X, Qin Y, Dai A, Zhang Y, Xue H, Ni H, *et al*. SMAD4 is involved in the development of endotoxin tolerance in microglia. *Cell Mol Neurobiol*. (2016) 36(5):777-88. doi: 10.1007/s10571-015-0260-0.<br>36. Lyn-Kew K, Rich E, Zeng X, Wen H, Kunkel SL, Newstead MW, *et al*. IRAK-M regulates chromatin remodeling in lung macrophages during experimental sepsis. *PLoS One*. (2010) 5(6):e11145. doi: 10.1371/journal.pone.0011145.<br>37. Nechama M, Kwon J, Wei S, Kyi AT, Welner RS, Ben-Dov IZ, *et al*. The IL-33-PIN1-IRAK-M axis is critical for type 2 immunity in IL-33-induced allergic airway inflammation. *Nat Commun*. (2018) 9(1):1603. doi: 10.1038/s41467-018-03886-6.

38. Petzl W, Gunther J, Pfister T, Sauter-Louis C, Goetze L, von Aulock S, *et al*. Lipopolysaccharide pretreatment of the udder protects against experimental *Escherichia coli* mastitis. *Innate Immun*. (2012) 18(3):467-77. doi: 10.1177/1753425911422407.
39. Rothschild DE, Zhang Y, Diao N, Lee CK, Chen K, Caswell CC, *et al*. Enhanced mucosal defense and reduced tumor burden in mice with the compromised negative regulator IRAK-M. *Ebiomedicine*. (2017) 15:36-47. doi: 10.1016/j.ebiom.2016.11.039.
40. Sano T, Izuishi K, Hossain MA, Inoue T, Kakinoki K, Hagiike M, *et al*. Hepatic preconditioning using lipopolysaccharide: Association with specific negative regulators of the Toll-like receptor 4 signaling pathway. *Transplantation*. (2011) 91(10):1082-9. doi: 10.1097/TP.0b013e31821457cb.
41. Shao B, Kitchens RL, Munford RS, Rogers TE, Rockey DC, Varley AW. Prolonged hepatomegaly in mice that cannot inactivate bacterial endotoxin. *Hepatology*. (2011) 54(3):1051-62. doi: 10.1002/hep.24488.
42. Steiger S, Kumar SV, Honarpisheh M, Lorenz G, Gunthner R, Romoli S, *et al*. Immunomodulatory molecule IRAK-M balances macrophage polarization and determines macrophage responses during renal fibrosis. *J Immunol*. (2017) 199(4):1440-52. doi: 10.4049/jimmunol.1601982.
43. Szatanik M, Hong E, Ruckly C, Ledroit M, Giorgini D, Jopek K, *et al*. Experimental meningococcal sepsis in congenic transgenic mice expressing human transferrin. *PLoS One*. (2011) 6(7):e22210. doi: 10.1371/journal.pone.0022210.
44. van der Windt GJ, Blok DC, Hoogerwerf JJ, Lammers AJ, de Vos AF, van't Veer C, *et al*. Interleukin 1 receptor-associated kinase M impairs host defense during *Pneumococcal Pneumonia*. *J Infect Dis*. (2012) 205(12):1849-57. doi: 10.1093/infdis/jis290.
45. Villar J, Cabrera NE, Casula M, Flores C, Valladares F, Díaz-Flores L, *et al*. Mechanical ventilation modulates TLR4 and IRAK-3 in a non-infectious, ventilator-induced lung injury model. *Respir Res*. (2010) 11(1):27. doi: 10.1186/1465-9921-11-27.
46. Xia Q, Zhou Y, Wang X, Fu S. Interleukin-1 receptor-associated kinase 3 downregulation in peripheral blood mononuclear cells attenuates immunosuppression in sepsis. *Exp Ther Med*. (2018) 15(2):1586-93. doi: 10.3892/etm.2017.5549.
47. Xiong Y, Medvedev AE. Induction of endotoxin tolerance in vivo inhibits activation of IRAK4 and increases negative regulators IRAK-M, SHIP-1, and A20. *J Leukoc Biol*. (2011) 90(6):1141-8. doi: 10.1189/jlb.0611273.
48. Yu S, Liu X, Zhang N, Yang S, Mao C, Feng S, *et al*. Protection of lipopolysaccharide (LPS) preconditioning against endotoxin-induced uveitis (EIU) in rats is associated with overexpression of interleukin-1 receptor-associated kinase-M (IRAK-M). *Ocul Immunol Inflamm*. (2018) 26(6):943-950. doi: 10.1080/09273948.2017.1291842.
