# Supplementary material for: Analysis of interleukin-1 receptor associated kinase-3 (IRAK3) function in modulating expression of inflammatory markers in cell culture models: A systematic review and meta-analysis
Source: PLoS One. 2020 Dec 31;15(12):e0244570. doi: 10.1371/journal.pone.0244570 (PMC7774834; doi:10.1371/journal.pone.0244570)
Supplement: S2 File — (PDF) [file pone.0244570.s003.pdf]

SUPPLEMENTARY FIGURES

Study or Subgroup	Intervention(1-challenge) Mean	SD	Total	Control Mean	SD	Total	Weight	Std. Mean Difference IV, Random, 95% CI
**IRAK3 mRNA expression (Mouse primary cells) - ST**								
Cole (2012)	313	95	3	100	6	3	33.4%	2.53 [-0.39, 5.45]
Li (2009)	87	20	3	100	21	3	43.6%	-0.51 [-2.18, 1.17]
Srivastav (2015)	207	25	3	100	13	3	22.9%	4.30 [-0.15, 8.74]
**Subtotal (95% CI)**			**9**			**9**	**100.0%**	**1.61 [-1.25, 4.47]**
Heterogeneity: Tau² = 4.16; Chi² = 6.01, df = 2 (P = 0.05); I² = 67%								
Test for overall effect: Z = 1.10 (P = 0.27)								
**IRAK3 mRNA expression (Mouse primary cells) - IT**								
Cole (2012)	238	26	3	100	6	3	11.3%	5.85 [-0.02, 11.72]
Emal (2019)	460	30	4	100	15	4	4.9%	13.20 [4.02, 22.38]
Lagler (2009)	193	11	3	100	39	3	34.1%	2.60 [-0.38, 5.57]
Paik (2019)	590	120	4	100	20	4	24.9%	4.95 [1.28, 8.63]
Srivastav (2015)	314	31	3	100	13	3	7.9%	7.20 [0.07, 14.34]
Yee (2013)	1,660	380	3	100	91	3	17.0%	4.52 [-0.13, 9.16]
**Subtotal (95% CI)**			**20**			**20**	**100.0%**	**4.76 [2.68, 6.83]**
Heterogeneity: Tau² = 0.98; Chi² = 5.83, df = 5 (P = 0.32); I² = 14%								
Test for overall effect: Z = 4.49 (P < 0.00001)								
**IRAK3 mRNA expression (Mouse primary cells) - LT**								
Li (2009)	98	20	3	100	21	3	47.7%	-0.08 [-1.68, 1.52]
Rajaiah (2013)	242	8	3	100	2	3	18.0%	19.48 [0.60, 38.36]
Srivastav (2015)	590	57	3	100	13	3	34.3%	9.48 [0.19, 18.78]
**Subtotal (95% CI)**			**9**			**9**	**100.0%**	**6.73 [-3.40, 16.85]**
Heterogeneity: Tau² = 55.30; Chi² = 7.93, df = 2 (P = 0.02); I² = 75%								
Test for overall effect: Z = 1.30 (P = 0.19)								

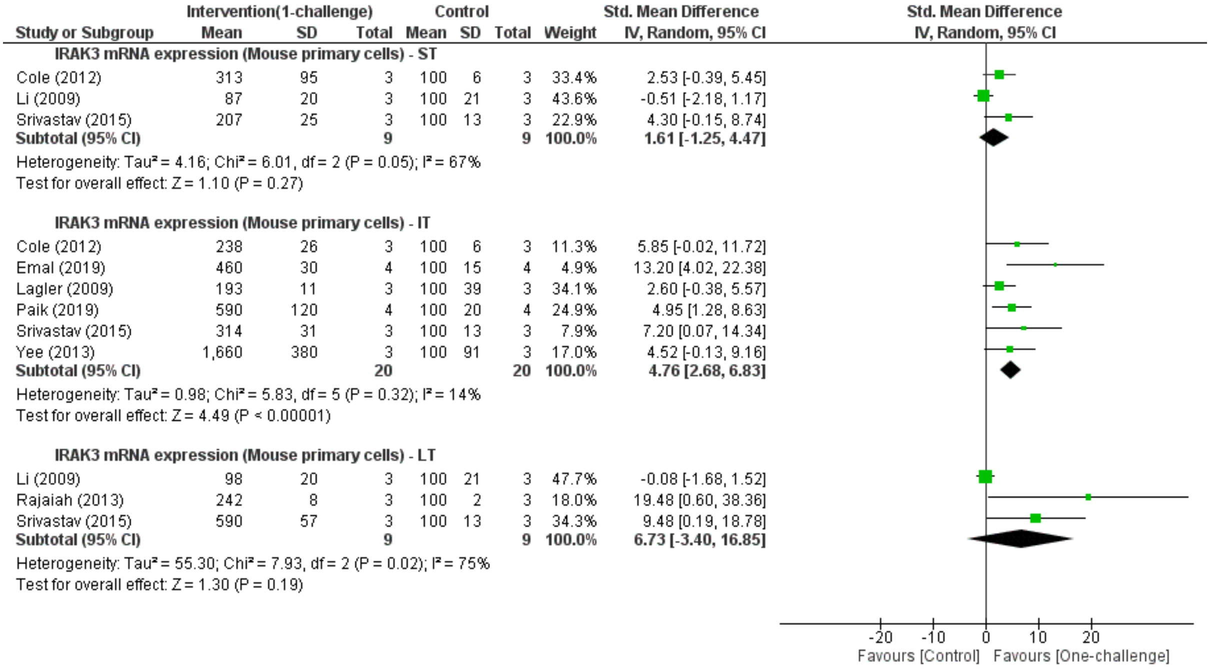

S1 Fig. IRAK3 mRNA expression in mouse primary cells: control group versus one-challenge intervention group. Intervention was performed using LPS [1-6], *L. donovani* [7] or *S. pneumoniae* [8]. IRAK3 mRNA expression was measured at short term (ST; 5min – 3h), intermediate term (IT; 4h – 15h) and long term (LT; 16h – 48h) after one-challenge intervention.

Study or Subgroup	Intervention(1-challenge) Mean	SD	Total	Control Mean	SD	Total	Weight	Std. Mean Difference IV, Random, 95% CI
**NF-kB reporter gene expression (Cell lines) - LT (6h - 24h)**								
Han (2008)	238.11	9.52	3	100	8.57	3	2.6%	12.20 [0.31, 24.09]
Kim (2008)	850	225	3	100	2.5	3	21.5%	3.77 [-0.21, 7.75]
Srivastav (2015)	142.86	12.5	3	100	7.14	3	25.4%	3.37 [-0.26, 6.99]
Wesche (1999)	233.61	57.96	3	100	57.96	3	50.5%	1.84 [-0.55, 4.24]
**Subtotal (95% CI)**			**12**			**12**	**100.0%**	**2.92 [0.98, 4.86]**
Heterogeneity: Tau² = 0.45; Chi² = 3.34, df = 3 (P = 0.34); I² = 10%								
Test for overall effect: Z = 2.95 (P = 0.003)								

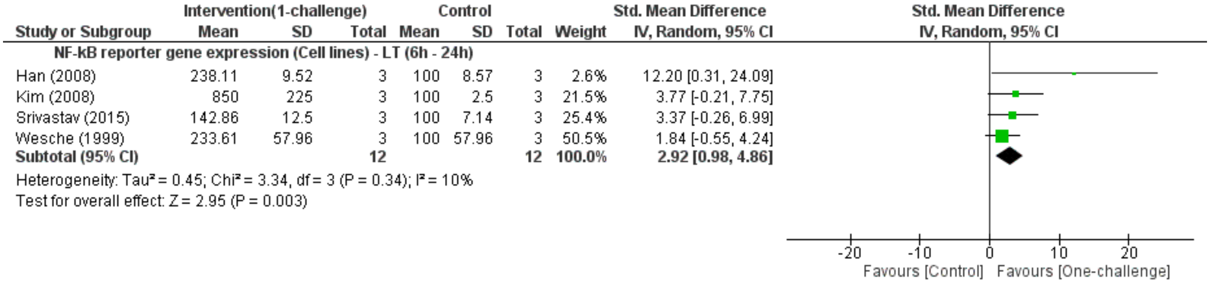

S2 Fig. NF-κB activation outcome for cell lines: control group versus one-challenge intervention group. Intervention was performed using LPS [9-12] or *L. donovani* [7]. NF-κB activity was measured at long term (LT_N; 6h – 24h) after one-challenge intervention.

Study or Subgroup	Intervention(1-challenge) Mean	SD	Total	Control Mean	SD	Total	Weight	Std. Mean Difference IV, Random, 95% CI
**TNF-a protein expression (Cell lines) - IT**								
Brudecki (2013)	2,789.5	324	3	52	52	3	12.7%	9.44 [0.19, 18.69]
Du (2014)	543	44.86	4	128.57	28.58	4	16.1%	9.58 [2.85, 16.32]
Han (2008)	1,400	150	3	60	6.67	3	11.9%	10.10 [0.22, 19.98]
Kim (2008)	65,000	15,833	3	1	5,833	3	19.4%	4.36 [-0.14, 8.86]
Kim (2012)	2,631.58	286.5	3	97.74	14.38	3	12.0%	9.99 [0.21, 19.77]
Mandrekar (2009)	15,000	880	3	200	50	3	5.4%	19.00 [0.58, 37.41]
Srivastav (2015)	33.34	3.12	3	35.42	2.92	3	22.5%	-0.55 [-2.24, 1.14]
**Subtotal (95% CI)**			**22**			**22**	**100.0%**	**6.89 [1.99, 11.79]**
Heterogeneity: Tau² = 26.99; Chi² = 24.81, df = 6 (P = 0.0004); I² = 76%								
Test for overall effect: Z = 2.76 (P = 0.006)								
**TNF-a protein expression (Cell lines) - LT**								
Al-Shaghdali (2019)	3,187.5	156.25	3	0	1	3	1.8%	23.08 [0.74, 45.42]
Daskalaki (2019)	116,666	11,545.85	3	76.92	5.33	3	5.5%	11.42 [0.28, 22.57]
Domon (2008)	20,000	1,000	3	1,000	10	3	2.0%	21.49 [0.68, 42.31]
Ferlito (2001)	200	57.73	3	20	10	3	13.8%	3.48 [-0.24, 7.19]
Hajshengallis (2004)	1,012	176	3	9	7	3	10.0%	6.44 [0.02, 12.87]
Hassan (2009)	12,880	1,630	3	750	200	3	7.9%	8.36 [0.13, 16.58]
Kim (2012)	3,292.31	532.93	3	100	13.32	3	9.6%	6.77 [0.04, 13.51]
Nakayama (2004)	18,690	2,190	3	160	160	3	6.9%	9.55 [0.19, 18.90]
Parmar (2018)	1,153.85	76.92	3	38.46	1	3	3.2%	16.40 [0.48, 32.32]
Peck (2004)	3,070	1,229.76	3	150	13.86	3	14.7%	2.69 [-0.36, 5.73]
Srivastav (2015)	39.59	3.54	3	35.42	2.92	3	16.0%	1.03 [-0.86, 2.91]
Sun (2014)	1,636.36	90.91	5	81.82	9.09	5	4.8%	21.73 [9.44, 34.03]
Sung (2013)	1,632.35	98.24	3	45	9.89	3	2.7%	18.19 [0.55, 35.82]
Xiong (2011)	4,608.7	152.52	3	434.78	17.39	3	1.1%	30.76 [1.01, 60.51]
**Subtotal (95% CI)**			**44**			**44**	**100.0%**	**7.53 [4.35, 10.71]**
Heterogeneity: Tau² = 15.52; Chi² = 36.09, df = 13 (P = 0.0006); I² = 64%								
Test for overall effect: Z = 4.64 (P < 0.00001)								
**IL-6 protein expression (Cell lines) - IT**								
Brudecki (2013)	3,450	222	3	66	66	3	20.1%	16.53 [0.49, 32.57]
Du (2014)	913.33	93.32	4	16.66	1.6	4	76.0%	11.81 [3.57, 20.06]
Liu (2016)	1,765.25	21.74	3	986.96	8.7	3	3.9%	37.60 [1.26, 73.95]
**Subtotal (95% CI)**			**10**			**10**	**100.0%**	**13.77 [6.58, 20.96]**
Heterogeneity: Tau² = 0.00; Chi² = 1.98, df = 2 (P = 0.37); I² = 0%								
Test for overall effect: Z = 3.75 (P = 0.0002)								
**IL-6 protein expression (Cell lines) - LT**								
Al-Shaghdali (2019)	254.55	34.1	3	6.81	1	3	10.9%	8.22 [0.12, 16.31]
Domon (2008)	1,950	190	3	1,000	10	3	17.2%	5.65 [-0.04, 11.33]
Hajshengallis (2004)	302	93	3	11	5	3	25.2%	3.53 [-0.23, 7.30]
Ho (2020)	1,392.9	787.27	6	142.9	87.45	6	36.2%	2.06 [0.55, 3.57]
Parmar (2018)	735.29	58.82	3	41.18	1	3	5.1%	13.35 [0.36, 26.34]
Peck (2004)	237	15.59	3	25	1.73	3	4.0%	15.29 [0.44, 30.14]
Sung (2013)	2,253.31	82.24	3	65.44	23.03	3	1.2%	28.98 [0.95, 57.02]
Twayana (2019)	2,642.9	151.32	2	107	25.31	2	0.2%	13.36 [-62.24, 88.96]
**Subtotal (95% CI)**			**26**			**26**	**100.0%**	**5.17 [2.03, 8.31]**
Heterogeneity: Tau² = 6.49; Chi² = 12.41, df = 7 (P = 0.09); I² = 44%								
Test for overall effect: Z = 3.23 (P = 0.001)								

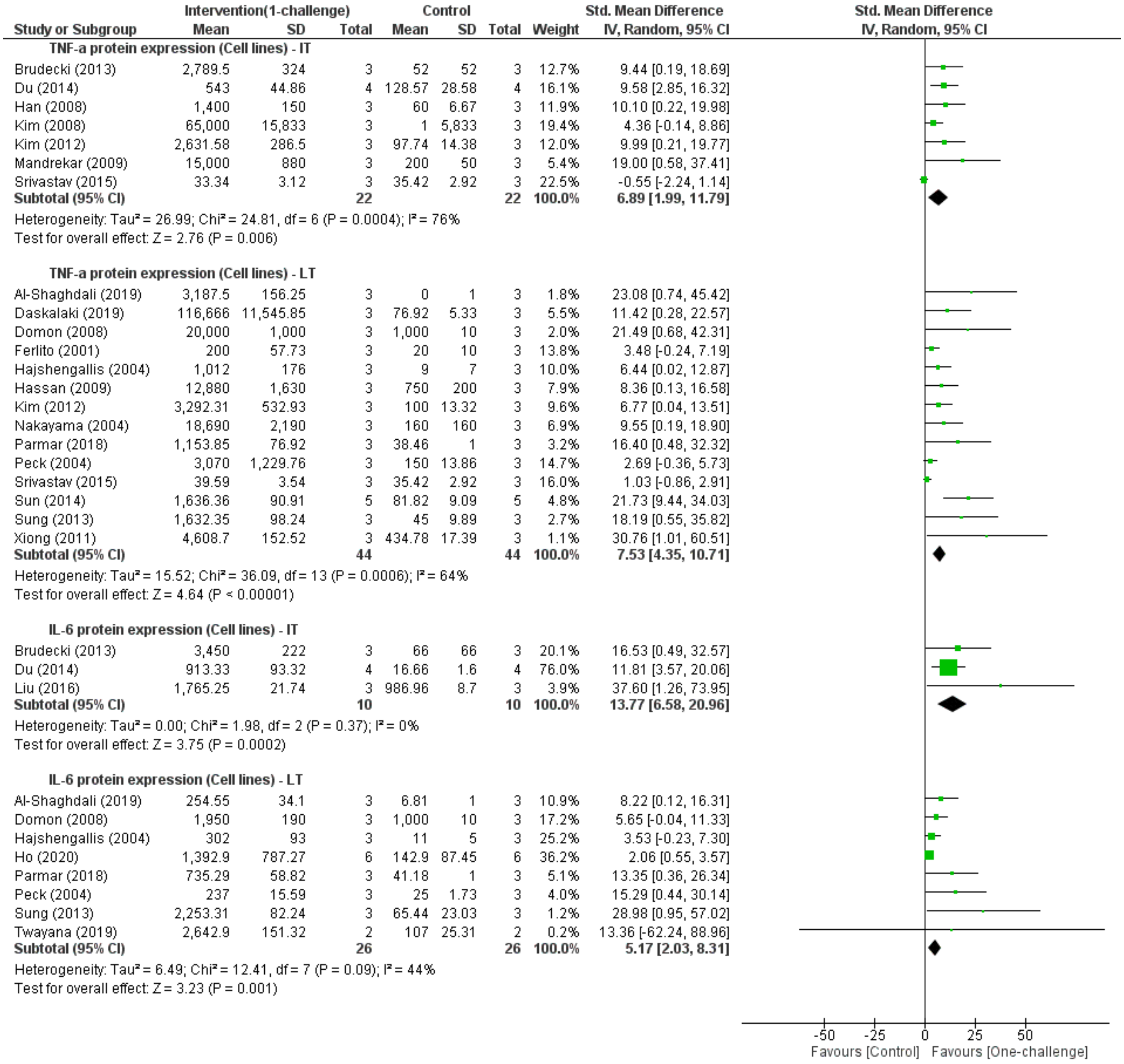

S3 Fig. TNF-α and IL-6 protein expression outcome for cell lines: control group versus one-challenge intervention group. Intervention was performed using LPS [9-11, 13-27], Pam3CSK4 [28], peptidoglycan [29] or *L. donovani* [7]. TNF-α and IL-6 protein levels were measured at intermediate term (IT; 4h – 15h) and long term (LT; 16h – 48h) after one-challenge intervention.

References

1. Cole TS, Zhang M, Standiford TJ, Newstead M, Luther J, Zhang J, et al. IRAK-M modulates expression of IL-10 and cell surface markers CD80 and MHC II after bacterial re-stimulation of tolerized dendritic cells. Immunol Lett. 2012;144(1-2):49-59.
2. Li Y, Annette EH, Lagoo AS, Kuchibhatla M, Pan H, Cohen HJ, et al. Differential gene expression of interleukin-1 receptor associated kinase-1 and interleukin-1 receptor associated kinase-M in peripheral blood mononuclear cells of young and aged rats following preconditioning with endotoxin. Shock. 2009;31(1):55-63.
3. Rajaiah R, Perkins DJ, Polumuri SK, Zhao A, Keegan AD, Vogel SN. Dissociation of endotoxin tolerance and differentiation of alternatively activated macrophages. J Immunol. 2013;190(9):4763-72.
4. Yee NK, Hamerman JA. β(2) integrins inhibit TLR responses by regulating NF-κB pathway and p38 MAPK activation. Eur J Immunol. 2013;43(3):779-92.
5. Paik S, Choe JH, Choi GE, Kim JE, Kim JM, Song GY, et al. Rg6, a rare ginsenoside, inhibits systemic inflammation through the induction of interleukin-10 and microRNA-146a. Sci Rep. 2019;9(1):4342.
6. Emal D, Rampanelli E, Claessen N, Bemelman FJ, Leemans JC, Florquin S, et al. Calcineurin inhibitor Tacrolimus impairs host immune response against urinary tract infection. Sci Rep. 2019;9(1):106.
7. Srivastav S, Saha A, Barua J, Ukil A, Das PK. IRAK-M regulates the inhibition of TLR-mediated macrophage immune response during late *in vitro Leishmania donovani* infection. Eur J Immunol. 2015;45(10):2787-97.
8. Lagler H, Sharif O, Haslinger I, Matt U, Stich K, Furtner T, et al. TREM-1 activation alters the dynamics of pulmonary IRAK-M expression *in vivo* and improves host defense during *Pneumococcal Pneumonia*. J Immunol. 2009;183(3):2027-36.
9. Domon H, Honda T, Oda T, Yoshie H, Yamazaki K. Early and preferential induction of IL-1 receptor-associated kinase-M in THP-1 cells by LPS derived from *Porphyromonas gingivalis*. J Leukoc Biol. 2008;83(3):672-9.
10. Han GK, Kim NR, Min GG, Jung ML, Seung YL, Mi YK, et al. Lipoteichoic acid isolated from *Lactobacillus plantarum* inhibits lipopolysaccharide-induced TNF-α production in THP-1 cells and endotoxin shock in mice. J Immunol. 2008;180(4):2553-61.
11. Kim YI, Park JE, Martinez-Hernandez A, Yi AK. CpG DNA prevents liver injury and shock-mediated death by modulating expression of interleukin-1 receptor-associated kinases. J Biol Chem. 2008;283(22):15258-70.
12. Wesche H, Gao X, Li X, Kirschning CJ, Stark GR, Cao Z. IRAK-M is a novel member of the pelle/interleukin-1 receptor-associated kinase (IRAK) family. J Biol Chem. 1999;274(27):19403-10.
13. Brudecki L, Ferguson DA, McCall CE, El Gazzar M. Mitogen-activated protein kinase phosphatase 1 disrupts proinflammatory protein synthesis in endotoxin-adapted monocytes. Clin Vaccine Immunol. 2013;20(9):1396-404.
14. Du J, Nicolaes GA, Kruijswijk D, Versloot M, van der Poll T, van 't Veer C. The structure function of the death domain of human IRAK-M. Cell Commun Signal. 2014;12:77.
15. Ferlito M, Romanenko OG, Ashton S, Squadrito F, Halushka PV, Cook JA. Effect of cross-tolerance between endotoxin and TNF-α or IL-1β on cellular signaling and mediator production. J Leukoc Biol. 2001;70(5):821-9.
16. Hajishengallis G, Sojar H, Genco RJ, DeNardin E. Intracellular signaling and cytokine induction upon interactions of *Porphyromonas gingivalis* fimbriae with pattern-recognition receptors. Immunol Invest. 2004;33(2):157-72.
17. Kim CH, Kim GH, Kim JY, Kim NR, Jung BJ, Jeong JH, et al. Probiotic genomic DNA reduces the production of pro-inflammatory cytokine tumor necrosis factor-alpha (TNF-α) FEMS Microbiol Lett. 2012;328(1):13-9.

18. Liu X, Qin Y, Dai A, Zhang Y, Xue H, Ni H, et al. SMAD4 is involved in the development of endotoxin tolerance in microglia. Cell Mol Neurobiol. 2016;36(5):777-88.
19. Mandrekar P, Bala S, Catalano D, Kodys K, Szabo G. The opposite effects of acute and chronic alcohol on lipopolysaccharide-induced inflammation are linked to IRAK-M in human monocytes. J Immunol. 2009;183(2):1320-7.
20. Peck OM, Williams DL, Breuel KF, Kalbfleisch JH, Fan H, Tempel GE, et al. Differential regulation of cytokine and chemokine production in lipopolysaccharide-induced tolerance and priming. Cytokine. 2004;26(5):202-8.
21. Sun Y, Li H, Sun MJ, Zheng YY, Gong DJ, Xu Y. Endotoxin tolerance induced by lipopolysaccharides derived from *Porphyromonas gingivalis* and *Escherichia coli*: Alternations in toll-like receptor 2 and 4 signaling pathway. Inflammation. 2014;37(1):268-76.
22. Sung NY, Yang MS, Song DS, Kim JK, Park JH, Song BS, et al. Procyanidin dimer B2-mediated IRAK-M induction negatively regulates TLR4 signaling in macrophages. Biochem Biophys Res Commun. 2013;438(1):122-8.
23. Xiong Y, Qiu F, Piao W, Song C, Wahl LM, Medvedev AE. Endotoxin tolerance impairs IL-1 receptor-associated kinase (IRAK) 4 and TGF-β-activated kinase 1 activation, K63-linked polyubiquitination and assembly of IRAK1, TNF receptor-associated factor 6, and IκB kinase-γ and increases A20 expression. J Biol Chem. 2011;286(10):7905-16.
24. Al-Shaghdali K, Durante B, Hayward C, Beal J, Foey A. Macrophage subsets exhibit distinct *E. coli*-LPS tolerisable cytokines associated with the negative regulators, IRAK-M and Tollip. PLos One. 2019;14(5):e0214681.
25. Daskalaki MG, Vyrla D, Harizani M, Doxaki C, Eliopoulos AG, Roussis V, et al. Neorogioltriol and related diterpenes from the red alga *Laurencia* inhibit inflammatory bowel disease in mice by suppressing M1 and promoting M2-like macrophage responses. Mar Drugs. 2019;17(2).
26. Parmar N, Chandrakar P, Vishwakarma P, Singh K, Mitra K, Kar S. *Leishmania donovani* exploits Tollip, a multitasking protein, to impair TLR/IL-1R signaling for its survival in the host. J Immunol. 2018;201(3):957-70.
27. Ho SW, El-Nezami H, Shah NP. The protective effects of enriched citrulline fermented milk with *Lactobacillus helveticus* on the intestinal epithelium integrity against *Escherichia coli* infection. Sci Rep. 2020;10(1):499.
28. Hassan F, Islam S, Tumurkhuu G, Dagvadorj J, Naiki Y, Komatsu T, et al. Involvement of interleukin-1 receptor-associated kinase (IRAK)-M in toll-like receptor (TLR) 7-mediated tolerance in RAW 264.7 macrophage-like cells. Cell Immunol. 2009;256(1-2):99-103.
29. Nakayama K, Okugawa S, Yanagimoto S, Kitazawa T, Tsukada K, Kawada M, et al. Involvement of IRAK-M in peptidoglycan-induced tolerance in macrophages. J Biol Chem. 2004;279(8):6629-34.
